# A New High-Throughput-Screening-Assay for Photoantimicrobials Based on EUCAST Revealed Unknown Photoantimicrobials in Cortinariaceae

**DOI:** 10.3389/fmicb.2021.703544

**Published:** 2021-08-05

**Authors:** Johannes Fiala, Harald Schöbel, Pamela Vrabl, Dorothea Dietrich, Fabian Hammerle, Desirée Josefine Artmann, Ronald Stärz, Ursula Peintner, Bianka Siewert

**Affiliations:** ^1^Department of Pharmacognosy, Institute of Pharmacy, University of Innsbruck, Innsbruck, Austria; ^2^Institute of Microbiology, University of Innsbruck, Innsbruck, Austria; ^3^MCI - The Entrepreneurial School, Innsbruck, Austria

**Keywords:** photoantimicrobials, PACT, EUCAST, Cortinariaceae, anthraquinone, LED-technique

## Abstract

Antimicrobial resistance is one of the biggest health and subsequent economic threat humanity faces. Next to massive global awareness campaigns, governments and NGOs alike stress the need for new innovative strategies to treat microbial infections. One of such innovative strategies is the photodynamic antimicrobial chemotherapy (PACT) in which the synergistic effects of photons and drugs are exploited. While many promising reports are available, PACT – and especially the drug-design part behind – is still in its infancy. Common best-practice rules, such as the EUCAST or CLSI protocols for classic antibiotics as well as high-throughput screenings, are missing, and this, in turn, hampers the identification of hit structures. Hit-like structures might come from synthetic approaches or from natural sources. They are identified via activity-guided synthesis or isolation strategies. As source for new antimicrobials, fungi are highly ranked. They share the same ecological niche with many other microbes and consequently established chemical strategies to combat with the others. Recently, in members of the Cortinariaceae, especially of the subgenus *Dermocybe*, photoactive metabolites were detected. To study their putative photoantimicrobial effect, a photoantimicrobial high-throughput screening (HTS) based on The European Committee on Antimicrobial Susceptibility Testing (EUCAST) was established. After validation, the established HTS was used to evaluate a sample set containing six colorful representatives from the genus *Cortinarius* (i.e., *Cortinarius callisteus*, *C. rufo-olivaceus*, *C. traganus*, *C. trivialis*, *C. venetus*, and *C. xanthophyllus*). The assay is built on a uniform, light-emitting diode (LED)-based light irradiation across a 96-well microtiter plate, which was achieved by a pioneering arrangement of the LEDs. The validation of the assay was accomplished with well-known photoactive drugs, so-called photosensitizers, utilizing six distinct emission wavelengths (λ_exc_ = 428, 478, 523, 598, or 640 nm) and three microbial strains (*Candida albicans*, *Staphylococcus aureus*, and *Escherichia coli*). Evaluating the extracts of six *Cortinarius* species revealed two highly promising species, i.e., *C. rufo-olivaceus* and *C. xanthophyllus.* Extracts from the latter were photoactive against the Gram-positive *S. aureus* (*c* = 7.5 μg/ml, *H* = 30 J/cm^2^, λ = 478 nm) and the fungus *C. albicans* (*c* = 75 μg/ml, *H* = 30 J/cm^2^, λ = 478 nm).

## Introduction

Whenever microorganisms share the same ecological niche – as, for example, soil fungi and soil bacteria – an orchestra of chemical compounds evolves reaching from mediators of stimulative symbiosis to detrimental antibiosis (Frey-Klett et al., 2011; Deveau et al., 2018). Plenty of such natural products have commercial values, especially as pharmaceuticals (Hyde et al., 2019). For example, most antibiotics approved by the Food and Drug Administration (FDA) are natural products (Lewis, 2020) and belong to antibiosis, which is described as chemical warfare.

According to Künzler (2018), fungal cells usually defend themselves by secreting chemical effectors against microbial competitors, rather than by storing them intracellularly. Nevertheless, fruiting bodies – or more precisely the hyphae differentiating into fruit-body tissues – often contain promising antibiotics. For example, various antimicrobial triterpenoids were isolated from the fruiting bodies of polypores, especially of *Ganoderma* spp. (Dresch et al., 2015; Basnet et al., 2017). These observations are rather the rule than the exception because for most basidiomycete genera, an antimicrobial activity was found in extracts from the fruiting bodies. Just for a few genera, for example, *Cortinarius*, antimicrobial activities were infrequently described. This, however, contrasts with the observation that fruiting bodies of this genus are rarely infested by other microorganisms (Moser, 1972). Thus, we were wondering whether an important co-factor was missing in the common screening attempts.

Co-factors, which can influence the antimicrobial activity of a secondary metabolite, might be metals (Lachowicz et al., 2020), pH conditions (Lee et al., 1997; Wiegand et al., 2015), or just a spark of light (Wozniak and Grinholc, 2018; Dos Santos et al., 2019). Such light-activated defense strategies (Downum, 1992; Flors and Nonell, 2006) are well-known for members of the kingdom Plantae and were recently suggested to be also present in fungi (Siewert et al., 2019). Furthermore, light-activated natural compounds are promising pharmaceuticals (Hudson and Towers, 1991; Berenbaum, 1995; Siewert and Stuppner, 2019).

As part of a putative light-activated defense system, the first photosensitizers, i.e., light-activated chemical compounds, were recently activity-guided discovered in fruiting bodies of macromycota (Hammerle, 2021). Light-activated antimicrobial effects of basidiomycetes are, however, not described yet, despite promising hints (Siewert, 2021). The lack of described photoantimicrobials might be the consequence of a non-existing photo-antimicrobial high-throughput screening (HTS) assay.

In general, plenty of different antimicrobial susceptibility tests are available determining the minimal inhibitory activity (MIC) of a substance. The utilized techniques reach from diffusion over thin-layer chromatography to dilution methods (Balouiri et al., 2016). In recent years, two standard protocols – one published by the [CLSI (Weinstein and Lewis, 2020)] and the other by the European Committee on Antimicrobial Susceptibility Testing [EUCAST (Microbiology and Diseases, 2003)] – were established. Most promising for HTS assays are such microbroth-dilution assays, which are based on visual (CLSI) or spectrophotometric (EUCAST) turbidity measurements (Wiegand et al., 2008). Microbroth-dilution assays can be conducted in 96-well plates and, thus, allow a high throughput: Eight antibiotics can be tested in 10 different concentrations on one plate in the dark, including the sterility and growth controls (Wiegand et al., 2008).

The crucial part of every PhotoMIC assay is the irradiation. Nowadays, dental curing lights (Nielsen et al., 2015) or handmade LED setups (Morici et al., 2020) replaced previously used light bulbs and lasers (Calin and Parasca, 2009). Dental lights – originally designed to polymerize composite fillings – allow only single irradiation and, therefore, limit the throughput. The described LED setups (not limited to microbials) vary from a single-emitter LED (Ogonowska et al., 2019) over 24 (Hernández Quintanar et al., 2016) and 96 LEDs (Butler et al., 2010; Chen et al., 2012; Hopkins et al., 2016; Katz et al., 2018) to 195 (Bajgar et al., 2020) or even 432 diodes (Pieslinger et al., 2006). A drawback of all settings with less than 100 diodes is the missing homogenous light distribution throughout a 96-well plate (Chen et al., 2012; Hopkins et al., 2016; Hernández Quintanar et al., 2016; Ogonowska et al., 2019). Consequently, only parts of a 96-well plate can be used. Common to all multi-diode settings is the equidistant arrangement of the diodes along the printed circuit board. Taking the nature of light into account, however, we wondered whether an asymmetric positioning of the diodes might improve the all-over distribution of light. Having extrapolated simulations for single LEDs in mind, we hypothesized that a homogenous illumination with only 24 diodes is possible.

Here we will report on (1) the design of a modular, 24 LED-based irradiation setup for 96-well plates, (2) the establishment of a HTS-PhotoMIC assay, which was validated with five standard photosensitizers (PSs, curcumin, methylene blue (MB), phenalenone, rose bengal, and hypericin) and five irradiation wavelengths (λ = 428, 478, 523, 598, and 640 nm); and, (3) the results of a sample set existing out of six *Cortinarius* extracts and identifying the basidiomycetes *Cortinarius xanthophyllus* and *C. rufo-olivaceus* as species containing photoantimicrobial(s) active against *Staphylococcus aureus* and *Candida albicans.*

## Materials and Methods

### Optical Simulations, Irradiation Setup, and Light Measurements

The irradiation system is based on LED technology. To achieve uniform irradiance along the entire sample, the arrangement of the individual LEDs within the 6×4 LED array is crucial. Therefore, the LED positions were optimized and verified with optical simulations. The simulation is based on an optical model for single LEDs (Wood, 1994) and is modified to calculate irradiance distribution in terms of Cartesian coordinates (Moreno et al., 2006). To simulate the irradiance *E*(*x*, *y*, *z*) at any point of the *x*, *y*-plane at a working distance *z*, the 6×4 LED array is modeled as:

$$E\left(x,y,z\right)=\sum_{n=1}^{6}\sum_{m=1}^{4}\frac{z^{k}\cdot I_{0}}{{\left[{\left(x-x_{n}\right)}^{2}+{\left(y-y_{m}\right)}^{2}+z^{2}\right]}^{\frac{k+2}{2}}},$$

where *x_n_* and *y_m_* are the positions of the individual LEDs in meters, and *I*_0_ is the radiant intensity in Watt per steradian. The deviation of the manufactured LED from a perfect Lambertian emitter is considered with the correction factor *k*, which depends on the viewing angle θ_1/2_

$$k=-\frac{\ln2}{\ln\cos\theta_{\frac{1}{2}}}.$$

The viewing angle θ_1/2_ is the off-axis angle from the LED centerline where the radiant intensity is half of the peak value and is provided by the LED manufacturer. By varying the individual LED positions *x_n_* and *y_m_*, the irradiance distribution in the sample plane can be modified. To achieve a uniform irradiance distribution, the individual LED positions were optimized by a nonlinear least-square curve fitting method with constraints (Betts, 1976; Coleman and Li, 1994, 1996). Optical simulations and optimization were performed using Mathworks (2019).

**FIGURE 1:**
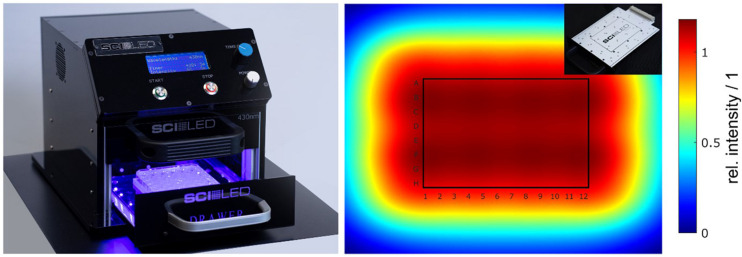
(Color online) Irradiation setup (SciLED) and simulated irradiance. The irradiation experiments were performed with a light-emitting diode (LED)-based setup (left). Due to its design, the LED modules can be easily exchanged to enable different wavelength settings (insert, top right). With the integrated user interface, the radiant exposure can be set by a timer and an intensity controller. The arrangement of the 6×4 LED array on the modules was optimized to achieve a uniform irradiance at the sample plane. Optical simulations of the irradiance showed a uniform distribution with a theoretical variation of less than 0.1% over the entire area of 96-well plates (right).

All irradiation experiments were carried out with an especially developed irradiation device (SciLED, MCI, Innsbruck) based on LED technology (Figure 1, left). The device consists of an extendable sample holder, where the 96-well plates can be inserted and reproducibly positioned in the irradiated area. If the experimental design requires alternative culture plates, e.g., Petri dishes, the sample holder can be easily adapted. To ensure a versatile area of application, the device has a modular design. Depending on the required irradiation conditions, the LED modular units (Figure 1, insert) can be exchanged. The LED modules were assembled with LUXEON CZ Color Line LEDs (Lumileds Holding, 2019). Each module consists of 24 LEDs of the same color (nominal peak wavelength). For this work, LEDs of the color violet (λ = 420–430 nm), blue (λ = 465–475 nm), green (λ = 520–540 nm), amber (λ = 585–600 nm), and red (λ = 624–634 nm) were used. The arrangement of the LEDs in the array was optimized to ensure a uniform irradiance. Figure 1 (right) shows the simulation of the irradiance of one LED modular unit. Next to the wavelength, the radiant exposure can be adjusted by a timer and an intensity controller.

Light measurements were conducted to characterize the illumination device. To check the uniformity, irradiance was measured using a radiometer and a chemical actinometer (i.e., ferrioxalate). Irradiance measurements were carried out along a 17 mm × 17 mm grid using the radiometer PM100D and the photodiode power sensor S120 VC with a measurement uncertainty of 3% (λ = 440–980 nm) and 5% (λ = 280–439 nm) (Thorlabs). The ferrioxalate actinometer (K_3_[Fe(C_2_O_3_)_3_]) and phenanthroline-based developing solutions were made using previously published methods (Hopkins et al., 2016). Spectral measurements were performed using the spectrometer MAYA 2000 Pro equipped with diffraction grating #HC-1 and an entrance slit of 5 μm (Ocean Insights), resulting in a spectral resolution of 0.66 nm FWHM. Light was coupled into the spectrometer via an optical fiber with a core diameter of 600 μm (QP600-1-SR-BX, Ocean Insights) and a cosine corrector (CC-3-UV-S, Ocean Insights). The spectrometer was calibrated with a wavelength calibration source (mercury–argon HG-2, Ocean Insights). To characterize the spectral power distribution, the peak shape was modeled with a sum of Gaussian functions (Reifegerste and Lienig, 2008; Supronowicz and Fryc, 2019). By fitting the sum of Gaussian functions to the spectral data, the wavelength where the intensity maximum occurs (peak wavelength in nm), the full width at half of the intensity maximum (FWHM in nm) and the full width at 10% of the intensity maximum (FW 0.1⋅*I*_*max*_ in nm) were calculated.

To evaluate the uniformity, the arithmetic mean irradiance *E_m_*, the standard deviation *SD*, and the coefficient of variation *cv* were calculated. As the uniformity was simulated and measured using a radiometer and a chemical actinometer, a comparison with the dimensionless parameter *cv* is convincing. The coefficient of variation is calculated as the ratio of the standard deviation and the arithmetic mean. Ensuring a precise representation of the spectral data by the model with Gaussian functions, the fit was accepted with a coefficient of determination *R*^2^ larger than 0.999.

### Mycochemical Part – Reagents, Instruments, and Methods

All solvents for the extraction and isolation processes were purchased from VWR International (Vienna, Austria). Acetone was distilled prior to use. Solvents for HPLC experiments had pro analysis (p.a.) quality and were obtained from Merck (Merck KGaA, Darmstadt, Germany). Ultrapure water was obtained with the Sartorius Arium^®^ 611 UV purification system (Sartorius AG, Göttingen, Germany).

Desiccation of the collected fungi was achieved with a Dörrex^®^ drying-apparatus from Stöckli (A. & J. Stöckli AG, Switzerland) operated at a temperature of 50°C. The fungal biomaterial was milled with a Bosch rotating coffee grinder MKM 6003 (Stuttgart, Germany). The samples were weighed with scales from KERN ALS 220-4 (KERN & SOHN GmbH, Balingen-Frommern, Germany) and Sartorius Cubis^®^-series (Sartorius AG, Göttingen, Germany). During the extraction process, the ultrasonic baths SONOREX RK 106, SONOREX RK 52, and SONOREX TK 52 (BANDELIN electronic GmbH & Co. KG, Berlin, Germany) were utilized. Vortexing was done with a Vortex-Genie 2 mixer (Scientific Industries, Inc., Bohemia, NY, United States). For centrifugation, an Eppendorf 5804R centrifuge with an F-45-30-11-30 place fixed angle rotor (Hamburg, Germany) was used.

Moreover, the HPLC-system Agilent Technologies 1200 Series with a binary pump, autosampler, column thermostat, and diode-array detector was used. HPLC systems were purchased from Agilent Technologies, Inc. (Santa Clara, CA, United States). For all HPLC measurements, a Synergi 4u MAX-RP 80-Å 150 mm × 4.60 mm column was used. HPLC-DAD-ESI-MS analysis was carried out with the modular system Agilent Technologies 1260 Infinity II equipped with a quaternary pump, vial sampler, column thermostat, diode-array detector, and an ion trap mass spectrometer (amaZon, Bruker, Bremen, Germany).

Pipetting was done with pipettes and tips from Eppendorf AG (Hamburg, Germany) and STARLAB International GmbH (Hamburg, Germany). Reagent reservoirs were obtained from Thermo Fisher Scientific (Waltham, MA, United States).

### Mycochemical Part

#### Preparation of Fungal Extracts

The fungal biomaterial (Supplementary Table 1) was dried on a desiccator (T∼50°C) right after collection and stored at room temperature until further use (*T* = 23.0°C, humidity = 20 ± 10%). The biomaterials were milled and sieved utilizing a mesh with the size of 400 μm. The extraction process was performed under light exclusion at room temperature. The powdered materials (*m* = 2.00 g) were extracted with acidified acetone (*V* = 20 ml, 0.1 v/v% 2N HCl) in an ultrasonic bath (*t* = 10 min). After centrifugation (*t* = 10 min, *T* = 4°C, *F* = 20,817 g), acetone was decanted and filtered through cotton wool. The fungal material was extracted twice more with acidified acetone (*V* = 5 ml). After centrifugation, the supernatant was collected, evaporated, and stored in brown glass vials at room temperature.

#### Reagents, Instruments, and Methods

Curcumin, dimethylsulfoxid (DMSO), lysogeny broth (LB) agar, phenalenone, and RPMI1640 medium were received from Merck KGaA (Darmstadt, Germany). Potato dextrose agar (PDA) and Mueller–Hinton broth (MHB) were purchased from VWR International (Vienna, Austria). Rose Bengal (RB) was received from TCI Europe (Zwijndrecht, Belgium). *Hypericum perforatum* extract (ethanol) was prepared from the pharmaceutical drug “Johanniskraut 600 mg forte” (Apomedica, Graz, Austria) by dissolving the filling of the film-coated tablet in ethanol after mechanical removal of the lactose. The 96-well plates (flat bottom) were bought from SARSTEDT (Nümbrecht, Germany).

The U-2001 spectrophotometer for adjusting the McFarland standard was from Hitachi (Chiyoda, Japan). For measurement of the 96-well plates, a Tecan Sunrise Remote Plate Reader (Tecan, Männedorf, Switzerland) was used. The adjustment of pH values was carried out with the pH-meter Mettler Toledo SevenMulti (Mettler-Toledo GmbH, Vienna, Austria).

#### Strains and Cultivation

All experiments on photodynamic inhibition (PDI) of growth of microorganisms (MOs) and the preparations were carried out under aseptic conditions in a laminar airflow cabinet at room temperature. The test strains used in this study were *Candida albicans* (501670), *Escherichia coli* (DSM1103), and *Staphylococcus aureus* (DSM1104). The strains were reactivated from frozen state and prepared according to the recommendations of the manufacturer^1^. Until further use, bacterial cultures were stored in darkness at 4°C on lysogeny broth agar. *C. albicans* was cultivated on potato dextrose agar under the same conditions.

For the PDI experiments, the stored cultures were reactivated, and an overnight culture was incubated (*T* = 37°C, *t* = 24 h, dark conditions). The bacterial culture inoculum was prepared using a spectrophotometer measurement at λ = 600 nm, as recommended by Nature Protocols (Wiegand et al., 2008). Turbidity was adjusted to a McFarland standard of 0.5 to prepare a standard suspension of 1.5 × 10^8^ colony-forming units (CFU)/ml. For yeast suspensions, turbidity was measured at λ = 530 nm, according to the EUCAST guidelines for antifungal susceptibility testing (Rodriguez-Tudela et al., 2008). Liquid media used for PDI experiments were MHB for bacteria and RPMI-1640 (double strength) for yeast.

#### Photo Minimal Inhibitory Concentration Assay

**FIGURE 2:**
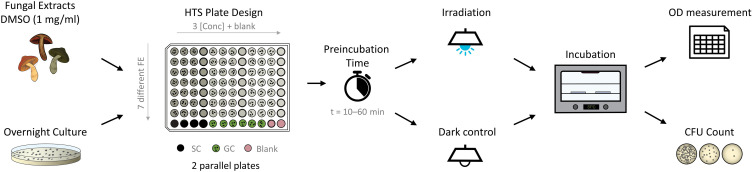
Flow chart of photo-antimicrobial (high-throughput screening; HTS) based on the microdilution method. The pipetting scheme represents the fast screening approach of extracts. Up to seven different fungal extracts with three concentrations each are tested on one plate. SC, sterility control; GC, growth control.

For the PDI experiments, flat-bottom 96-well plates were used. On each plate, an extract test section, growth control, fraction-blank, medium-blank, and sterility controls were set up (Figure 2). In the test section, three concentrations of fungal extracts (i.e., *c* = 25, 50, and 75 μg/ml), were tested. If needed, smaller or larger concentrations were tested as well. Furthermore, positive controls (dark condition) were established for each experiment: Curcumin (*c* = 30 μg/ml, 81.5 μM) for *C. albicans*, phenalenone (*c* = 75 μg/ml, 416.2 μM) for *E. coli*, and phenalenone (*c* = 25 μg/ml, 138.7 μM) for *S. aureus*.

Positive controls, fungal extracts (FE), and growth control were inoculated with an inoculum (*V* = 50 μl) of the test strains within *t* = 30 min after the turbidity adjustment. Two identical 96-well plates were prepared for both – dark and light – treatments. After 10 or 60 min of preincubation time in darkness, one plate was irradiated with the SciLED panel at λ = 478 nm for *t* = 19 min 8 s, corresponding to a light dose of *H* = 9.3 J/cm^2^. An alternative irradiation setup was *t* = 61 min 44 s, corresponding to a light dose of *H* = 30 J/cm^2^. The other plate was kept in a dark box at room temperature beside the SciLED for the time of irradiation. The dark control was handled as similar as possible to the irradiated sample.

After irradiation, both plates were submitted to turbidity measurements. Here, a plate reader was used, and before measuring the optical density (bacteria: λ = 600 nm, fungi: λ = 530 nm), the plates were shaken for 5 s. Viability controls were drawn from the control vials and plated on LB/PDA agar. Afterward, the 96-well plates and LB/PDA agar plates were incubated at *T* = 37°C in the dark for 24 h. A second measurement of turbidity was done, followed by taking samples of wells that showed inhibition (>20%) of population growth control.

Assessment of the PDI experiment was done by correlating the treated well to the uninhibited growth control. Turbidity of fraction-blank and medium-blank was subtracted from corresponding wells to eliminate deviation caused by darkening or bleaching of media and extracts. Each concentration of the fungal extracts, the positive control, and the growth control were measured at least in triplicates, using different wells and samples, thus, as biological triplicate.

### Singlet-Oxygen Detection via the Dimethyl Anthracene-Assay

To analyze the ability of the six fungal extracts (FE) to generate singlet oxygen after irradiation, the previously described dimethyl anthracene (DMA) assay and a previously characterized irradiation setup were employed (Siewert et al., 2019). As a first step, a DMA solution in ethanol (*c* = 1.4 mM) (L1) and a L-ascorbic acid solution in ultrapure water (*c* = 100 mM, pH = 7.0–7.4) (L2) were prepared. The fungal extracts were dissolved in DMSO (*c* = 1 mg/ml, FE) and subsequently mixed with the stock solutions (L1 and L2) as well as pure ethanol (L3) to obtain four test-solutions (*V* = 10 μl FE + 190 μl test-solution): (1) a pure ethanolic solution of the FE to observe photochemical changes of the extract due to the irradiation, (2) a mixture with DMA to detect singlet oxygen, (3) a mixture with DMA and the antioxidant L-ascorbic acid to prove that singlet oxygen caused the oxidation of DMA, and (4) a control consisting of an ethanolic solution of the extract and L-ascorbic acid to control, that no undesired reaction occurs. DMSO (*V* = 10 μl) was used as negative control and berberine (*c* = 1 mg/ml, 2.97 mM, DMSO, *V* = 10 μl) were used as positive controls. Thereafter, optical densities at the wavelengths λ = 377 nm and 468 nm were measured with a plate reader (*t* = 0 min), followed by four cycles of blue light (λ = 468 nm, 6.2 J/cm^2^, berberine = positive control). All measurements were done as technical duplicates. The results of the DMA assay were presented as the mean ± Standard Error. Differences between the relative singlet oxygen formation values were statistically evaluated using one-way ANOVA followed by the Bonferroni post-test, and *p* < 0.05 was considered to be significant.

## Results

### Uniform Irradiance and Irradiation Conditions

**FIGURE 3:**
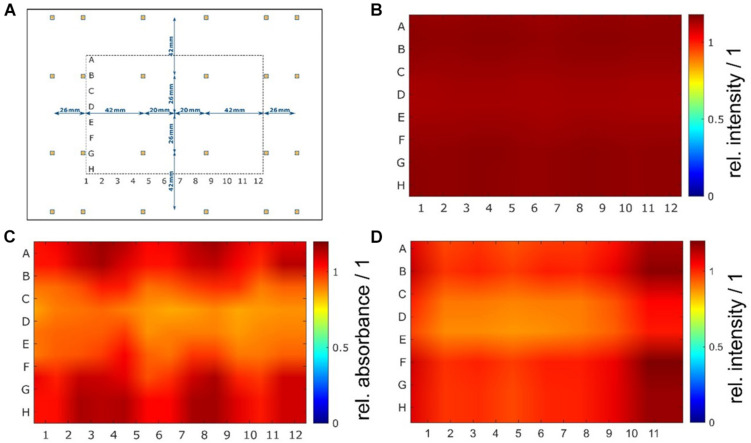
(Color online) Non-equidistant LED arrangement and results of homogeneity measurements. A nonlinear optimization of the individual LED positions in the *xy*-plane of the printed circuit board resulted in a symmetric but not equidistant arrangement with decreasing distances on the outside. For a better understanding of the LED arrangement, the position of the irradiated plate is illustrated as well **(A)**. The irradiance distribution at the sample plane was determined by optical simulations **(B)** and measured with a chemical actinometer **(C)** and a radiometer **(D)**. In the optical simulations, a very high uniformity with a theoretical coefficient of variation of less than 0.1% (*cv*_*sim*_ = 0.08%) was calculated. Experimental evaluation of the uniformity resulted in variations of less than 10% (chemical actinometer *cv*_*act*_ = 9% and radiometer *cv*_*rad*_ = 8%).

The nonlinear optimization of the individual LED positions in the array resulted in a symmetric but not equidistant arrangement. The objective was to achieve a homogenous irradiation distribution with theoretical variations below 5% in an irradiated area of 120 mm 90 mm (*x*×*y*), which approximately corresponds to the size of a 96-well plate. After several optimization steps, a calculated coefficient of variation *cv* = 0.08% was achieved in the optical simulations. Such uniformity was obtained by decreasing the relative spacing between the outside LEDs and positioning them beyond the area of the irradiated sample. The individual positions of the 24 LEDs are shown in Figure 3A. Experimental evaluation of the uniformity resulted in an actual variation between *cv* = 7% and *cv* = 8% for the irradiance measurements and a variation of *cv* = 9% for the chemical actinometer measurements. Over the entire area of a 96-well plate, the resulting irradiance distribution is homogeneous, and from the uniformity standpoint, all 96 wells can be used for irradiation tests. Results of the optimization and irradiance distribution within the 96-well plates are shown in Figures 3B–D.

To fully characterize the irradiation device, the spectral power distribution and irradiance were measured for every LED module (violet, blue, green, amber, and red). From these measurements, several spectral parameters, including the actual peak wavelength and the full width at half maximum, were calculated, and the average irradiance was determined. Spectral power distributions varied between 15 nm FWHM for violet LEDs and 33 nm FWHM for green LEDs. Average irradiance was the highest for the violet LED module with *E*_*m*_ = 13 ± 1.0 mW/cm^2^ and the lowest for the amber LED module with *E*_*m*_ = 1.1 ± 0.08 mW/cm^2^. All results on the irradiation conditions are reported in Table 1.

**TABLE 1:** Optical characterization of the irradiation device.

LED module color	Spectral information				Irradiance		
	Wavelength	FWHM	FW 0.1 ⋅*I*_*max*_	*R* ^2^	*E*_*m*_	*SD*	*cv*
	[nm]	[nm]	[nm]	[1]	[mW/cm^2^]	[mW/cm^2^]	[1]
Violet	428	15	36	0.9994	13	1.0	0.081
Blue	478	27	63	0.9991	8.7	0.70	0.076
Green	523	33	78	0.9998	6.0	0.44	0.073
Amber	598	16	38	0.9994	1.1	0.084	0.078
Red	640	18	45	0.9999	6.4	0.47	0.074

*The spectral power distributions and the irradiance at the sample plane were measured for all LED module colors. From the spectral data, the actual peak wavelength, the full width at half of the intensity maximum (FWHM), and the full width at 10% of the intensity maximum (FW 0.1⋅I_max_) were obtained by fitting a sum of Gaussian functions to the data. From the irradiance measurements, the arithmetic mean (E_m_), the standard deviation (SD) and the coefficient of variation (cv) were calculated.*

### Establishment of a High-Throughput Screening-Protocol

A high-throughput assay was developed based on the gold-standard microdilution method (Wiegand et al., 2008; Benkova et al., 2020). Like the classic method, the HTS started with an overnight culture of the selected test organisms (*E. coli*, *S. aureus*, and *C. albicans*) and, separately, with the test compounds or extracts of interest (Figure 2). In the next step, a stock solution of the test extracts or compounds was generated in DMSO and successively diluted in media. MHB was used for the bacteria, while the yeast was cultured in RPMI double strength. In Figure 2, a flow chart is displayed, including the pipetting scheme for the testing extracts. In Supplementary Figure 1, the respective flow chart with a pipetting plan for pure compounds is shown. In contrast to the classic microdilution assay, a blank of each tested compound was needed to avoid false-negative effects in the final OD reading to determine the MIC. The next step was a preincubation step, followed by an irradiation step with the chosen wavelength and light dose. A dark control was conducted in parallel to examine the effect of light. After the light treatment step (and the respective “dark treatment step”), the plates were incubated for *t* = 24 h. Finally, an OD measurement was performed to quantify the MIC, and – if needed, – the treated dilutions were submitted to a CFU count to determine the MBC.

### Establishment of the Photoantimicrobial Assay and Its Validation With Known Photosensitizers

**FIGURE 4:**
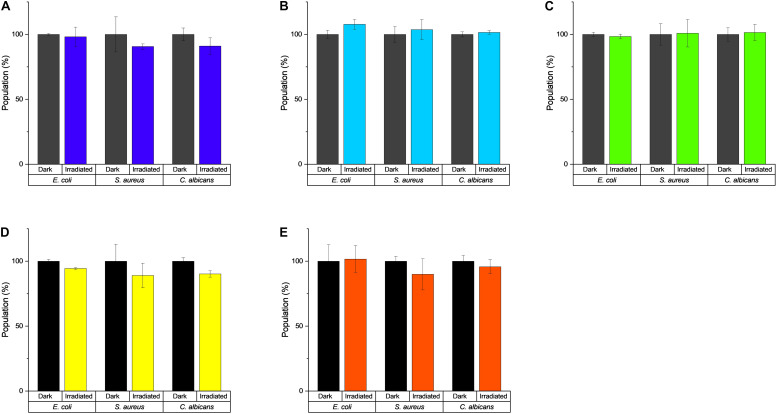
Effect of light irradiation on the growth of *E. coli*, *S. aureus*, and *C. albicans*. **(A)** λ_irr_ = 428 nm, *H* = 30 J/cm^2^, **(B)** λ_irr_ = 478 nm, *H* = 30 J/cm^2^, **(C)** λ_irr_ = 523 nm, *H* = 30 J/cm^2^, **(D)** λ_irr_ = 598 nm, H = 9.3 J/cm^2^, and **(E)** λ_irr_ = 640 nm, *H* = 30 J/cm^2^.

In the first step, the light tolerance of the test organisms (i.e., *E. coli*, *S. aureus*, and *C. albican*s) was examined. To achieve this, the microorganisms were irradiated utilizing the five different LED modules with light doses up to *H* = 30 J/cm^2^. Although a statistical analysis (see Supplementary Part 2.2) revealed a mathematical significance for the three groups (Supplementary Figure 2), the *de facto* decrease/increase of the population was very small (below 10%) and still within the range of biological variations (Figure 4). Therefore, all observed effects will be due to the combined effects of the light and the test compound/extract.

**FIGURE 5:**
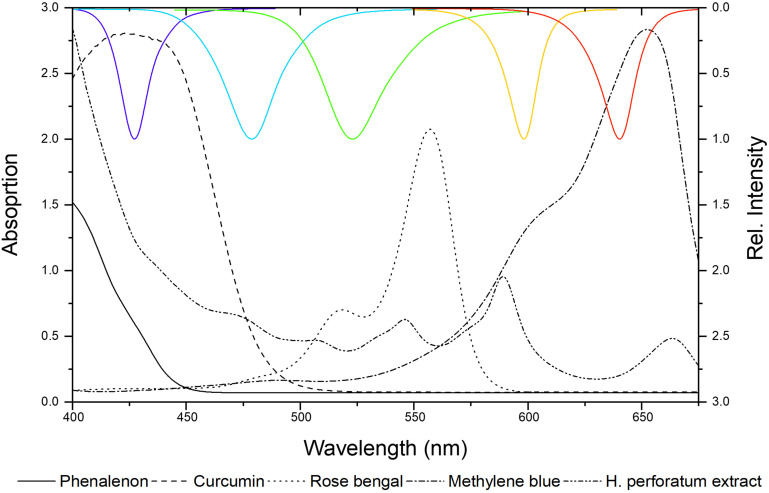
Absorbance spectra of positive controls (PCs) versus emission spectra of the LED modules (λ_irr_ = 428, 478, 523, 598, and 640 nm from left to right).

Next, well-established photosensitizers were selected. In detail, phenalenone, curcumin, rose bengal (RB), methylene blue (MB), and a *Hypericum perforatum* (HP) extract (photoactive ingredient: hypericin) were chosen to validate the irradiation setup. These positive controls (PCs) were characterized by absorption properties complementary to the LED-emission profiles (Figure 5). As depicted, several LED modules can activate individual PCs, as their absorbance bands fit more than one LED module. In Table 2, the PhotoMIC values – generated in accordance with the EUCAST guidelines – are given. For each LED module and tested microorganism, the most active PS is represented in Figure 5, although for several LED modules, a selection of PSs worked (see Supplementary Part Chapter 2.3). For example, the growth of *S. aureus* was not only impeded with yellow light (λ_irr_ = 523 nm, 30 J/cm^2^) and RB (*c* = 6 μg/ml, Table 2), but also with yellow light (λ_irr_ = 523 nm, 30 J/cm^2^) and HP (*c* = 150 μg/ml). The MIC using RB (*c* = 6 μg/ml), however, was more promising and is, thus, displayed in Table 2. The dose–response curves are depicted in Supplementary Figures 3–7.

**TABLE 2:** Overview of the minimal inhibition concentrations under irradiation (PhotoMIC) of the investigated positive controls regarding the three tested microorganisms (MOs).

	428 nm	478 nm	523 nm	598 nm	640 nm	Dark
*C. albicans* (yeast)	Curc 4 μg/ml (10.9 μM) 60 min 30 J/cm^2^	Curc 30 μg/ml (81.5 μM) 10 min 9.3 J/cm^2^	HP 50 μg/ml 10 min 30 J/cm^2^	HP 200 μg/ml 10 min 9.3 J/cm^2^	MB 2.5 μg/ml (7.8 μM) 60 min 30 J/cm^2^	AMP 0.2 μg/ml (0.2 μM)
*E. coli* (gram negative)	Curc 40 μg/ml (108.6 μM) 10 min 30 J/cm^2^	PN* 75 μg/ml (416.2 μM) 10 min 9.3 J/cm^2^	RB* 150 μg/ml (154.1 μM) 10 min 30 J/cm^2^	n.s.	n.s.	CAP 2 μg/ml (6.2 μM)
*S. aureus* (gram positive)	Curc 4 μg/ml (10.9 μM) 10 min 9.3 J/cm^2^	PN 25 μg/ml (138.7 μM) 10 min 9.3 J/cm^2^	RB* 4 μg/ml (4.1 μM) 10 min 30 J/cm^2^	HP 150 μg/ml 10 min 9.3 J/cm^2^	n.d.	ERY 1 μg/ml (1.3 μM)

In the table, for each photosensitizer (PS) is given the PhotoMIC value with the utilized preincubation time (PI) and light dose (H). The last column contains minimal inhibitory activities (MICs) of standard antibiotics without light-irradiation. Curc, curcumin; PN, phenalenone; RB, rose bengal; HP, Hypericum perforatum extract; *, other PS worked as well; n.d, not detected; n.s, not selective; CAP, chloramphenicol; AMP, amphotericin B; ERY, erythromycin. For a full discussion see Supplementary Chapter 3.

### Mycochemical Analysis of Selected *Cortinarius* Species

Based on their colorful appearance, the fruiting bodies of six different *Cortinarius* species (i.e., *Cortinarius callisteus*, *C. rufo-olivaceus*, *C. traganus*, *C. trivialis*, *C. venetus*, and *C. xanthophyllus*) were selected to evaluate our photo-antimicrobial assay (see Supplementary Table 1 for collection information). In the first step, the dried material was extracted, and the obtained extracts (see Table 3) were analyzed spectroscopically (UV-Vis, Supplementary Figure 8) as well as chromatographically, i.e., HPLC combined with several hyphenated detectors (i.e., DAD, FLD, ELSD, MS; see Supplementary Figures 9–11). The results showed that the extract of *C*. *xanthophyllus* is not only the most complex but also the most intensely colored one (Supplementary Figures 8, 12). In detail, five intense peaks were detected at λ = 254 nm (Supplementary Table 3). The absorption maxima of all peaks were recorded (see Supplementary Figure 13). For the two major peaks (t_r, Peak 4_ = 28.3 and t_r, Peak 5_ = 34.2 min), they equaled λ_max, Peak 4_ = 436 nm and λ_max, Peak5_ = 525 nm.

**TABLE 3:** Initial weight of biomaterial of *Cortinarius* species and yield of extracts.

	Biomaterial [mg]	Yield of extract [mg, (%)]	Visual appearance
*C. callisteus*	1,643.7	14.6 (0.9)	Light yellow, muddy
*C. rufo-olivaceus*	1,784.9	91.0 (5.1)	Dark red, dull
*C. traganus*	1,709.7	18.2 (1.1)	Dark yellow, muddy
*C. trivialis*	1,958.1	22.0 (1.1)	Dark yellow, greasy
*C. venetus*	1,944.0	26.7 (1.4)	Light yellow, greasy
*C. xanthophyllus*	1,051.8	26.6 (2.5)	Purple, earthy, powder

The mass spectrometric analysis revealed a mass of m/z = 283 [M-H]^–^ for Peak 4 and the chemical formula C_16_H_12_O_5_. Taking the characteristic fluorescence properties of Peak 4 (Supplementary Figure 9A) and the TLC work of Hofbauer (1983) into account, this peak was annotated as parietin and confirmed by comparison with an authentic sample (Supplementary Figure 14). Also, Peaks 2, 3, and 5 were characterized by anthraquinone-like absorption spectra (Supplementary Figure 13). The red shift of the absorption maxima (Δλ = 57–87 nm) of all three peaks [compared with Peak 4 (parietin)] indicated an extended chromophore and, thus, hinted toward dimeric AQ-like structures. While Peak 2 (t_r_ = 25.4 min) and Peak 3 (t_r_ = 26.9 min) were also detected in the extract of *C. rufo-olivaceus*, they were putatively assigned as rufoolivacin A and C (Gill and Steglich, 1987; Zhang et al., 2009; Gao et al., 2010), which was in accordance with their mass peak of m/z = 557.2 [M+H]^+^ (Supplementary Table 3). Peak 5 (m/z = 556.2 [M+H]^+^) was not assigned yet, but might be an oxidated derivative of phlegmacin (MW = 576.6 g/mol), which was described in *C. xanthophyllus* (Hofbauer, 1983). Plenty of dimeric anthraquinones are known from related Cortinariaceae (Gill and Steglich, 1987; Elsworth et al., 1999; Zhang et al., 2009; Gao et al., 2010); thus, this putative annotation seems reasonable. Further discussion of the metabolic profiles can be found in the Supplementary Part (Chapter 3.1).

### Singlet-Oxygen Detection Assay (Dimethyl Anthracene-Assay)

The obtained extracts were submitted to the recently developed singlet oxygen high-throughput assay (DMA-assay) (Siewert et al., 2019). Out of the six investigated extracts, two, namely, *C. xanthophyllus* and *C. rufo-olivaceus*, showed the ability to produce ^1^O_2_ after being irradiated with blue light (Table 4). *C. xanthophyllus* was the most active extract: Irradiated at λ = 468 ± 27 nm (24.8 J/cm^2^), the extract produced 187% singlet oxygen compared with the well-known photosensitizer phenalen-1-one (Schmidt et al., 1994; Espinoza et al., 2016). Hence, this extract originating from natural sources showed photosensitizing activity as promising as those of synthetic compounds, such as phenalene-1-one.

**TABLE 4:** Results of the dimethyl anthracene (DMA) assay (blue light irradiation relative to phenalene-1-one).

	Singlet oxygen production [%]	Standard deviation [%]
*C. callisteus*	3.1	1.7
*C. rufo-olivaceus*	49.6	2.5
*C. traganus*	0.2	1.1
*C. trivialis*	4.2	2.0
*C. venetus*	4.4	0.2
*C. xanthophyllus*	187.1	2.1

### *Cortinarius xanthophyllus* Contains Photoantimicrobials Active Against *Staphylococcus aureus* and *Candida albicans*

**FIGURE 6:**
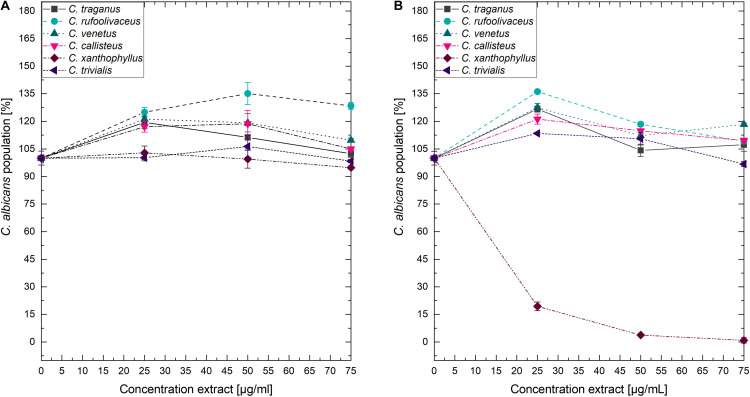
Dose–response curves of *C. albicans* treated with *Cortinarius* extracts. On the left side, **(A)** dark controls are shown. The right graph **(B)** represents the irradiated experiments (λ = 478 nm, H = 30 J/cm^2^, PI = 60 min).

**FIGURE 7:**
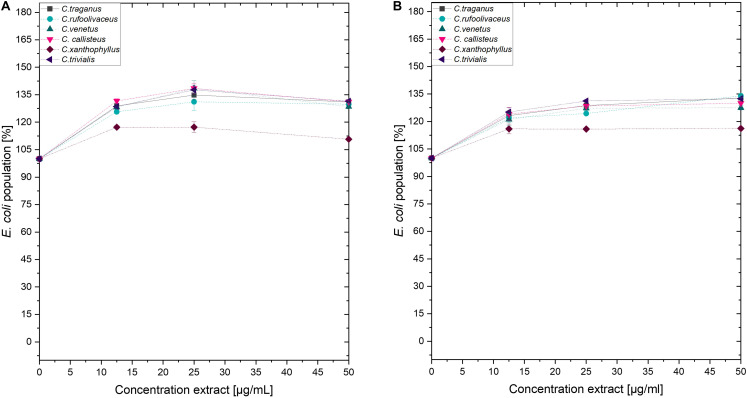
Dose–response curves of *E. coli* treated with *Cortinarius* extracts. On the left side, **(A)** dark controls are shown. The right graph **(B)** represents the irradiated experiments (λ = 478 nm, 30 J/cm^2^, PI = 60 min).

**FIGURE 8:**
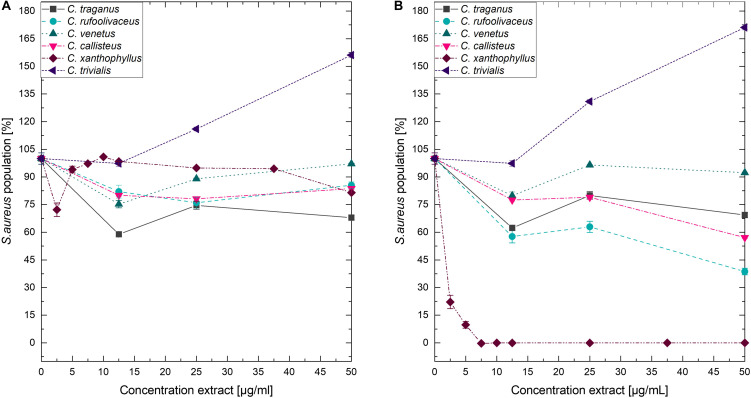
Dose–response curves of *S. aureus* treated with *Cortinarius* extracts. **(A)** Growth inhibition under dark conditions. **(B)** Growth inhibition under irradiation conditions (λ = 478 nm, H = 30 J/cm^2^, PI = 60 min).

Submitting all six extracts to the (photo)antimicrobial assay revealed that all extracts are inactive (*c* > 50 μg/ml) under the exclusion of light (Figures 6–8). Under light irradiation, however, the activity of the purple extract of *C. xanthophyllus* was significantly enhanced: The growth of the Gram-positive *S. aureus* (Figure 8) was completely inhibited with an extract concentration as low as *c* = 7.5 μg/ml and a light dose of *H* = 30 J/cm^2^ (λ = 478 nm). This also holds true for the photoactivity against the yeast *C. albicans*, where an extract concentration of *c* = 75 μg/ml (*H* = 30 J/cm^2^) led to complete growth inhibition (Figure 6). Against the gram-negative *E. coli*, however, *C. xanthophyllus* was inactive in the dark and under irradiation (Figure 7).

A weak enhancement of the growth inhibition effect was also seen for the *C. rufo-olivaceus* extract against *S. aureus* (IC_50_∼50 μg/ml). Nevertheless, this extract did not affect *C. albicans* under the tested conditions.

## Discussion

The hypothesis we wanted to test in the course of this study was that light is a neglected co-factor in antimicrobial screening assays. Therefore, a convenient HTS-screening assay based on the EUCAST protocol was established and validated. In the first step, an innovative LED-panel was invented achieving a homogenous irradiation of a 96-well plate. Finally, the hypothesis was tested with a set of six different *Cortinarius* species.

### The SciLED Irradiation System and Its Improved Uniform Irradiance Distribution

To achieve the first objective – the homogenous irradiation of a 96-well plate – the distance and number of the LEDs were optimized by simulations until the coefficient of variation, *cv*, was estimated to be less than 1%. Irradiance measurements and chemical actinometer measurements (Figure 3) confirmed the homogeneous irradiance. Nevertheless, the actual coefficient of variation from irradiance measurements was in the range between 7 and 9%. Deviations between the simulation and the measurement can be attributed to (i) differences between the modeled and the actual radiant intensity distribution of the LED, (ii) variations in the radiometric power of individual LEDs, and (iii) measurement uncertainties.

**FIGURE 9:**
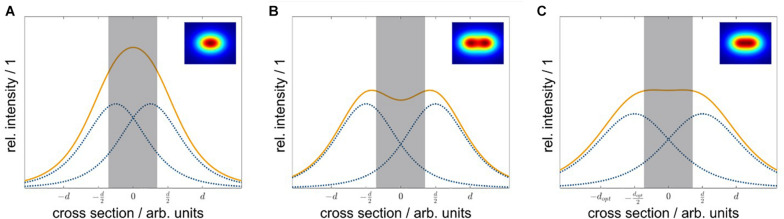
Side-view of the irradiance distribution depending on the LED distance. Depending on the LED distance ***d***, the resulting irradiance from the two overlapping LED irradiance distributions at the sample plane differs. Individual LED irradiance distributions are represented as dotted lines (blue), and the resulting distributions are shown as solid lines (orange) the gray bar indicates the maximum width of the sample plate. If the LED distance ***d*** is too low, the overlap in the center is high, and the resulting irradiance is enhanced **(A)**. For a too large LED distance ***d***, the overlap is small, and the resulting irradiance is reduced **(B)**. Using the optimal LED distance ***d*_*opt*_**, each LED contributes the right amount and the resulting irradiance in the center is constant **(C)**.

Although the real uniformity (*cv* = 8%) of the irradiation system with non-equidistant LED arrangement presented in this work was less than the expected uniformity from the simulation (*cv*_*sim*_ = 0.08%), the achieved homogeneity over the whole sample area was still significantly better compared with equidistant LED positioning. An optical simulation of a 6×4 LED array with an equidistant arrangement (△*x* = 35 mm, △*y* = 35 mm) resulted in a less homogeneous irradiance distribution (*cv*_*sim*_ = 5%) compared with the existing non-equidistant arrangement (*cv*_*sim*_ = 0.08%). To understand the positive effects of a non-equidistant positioning, the non-uniform radiant intensity distribution of LEDs must be considered: For one LED, the resulting irradiance distribution on the irradiated plane will show a non-linear decrease, with an increased distance from the beam center. For two LEDs, which are placed with a certain distance *d* next to each other, parts of the irradiance will overlap. Due to the superposition principle, the resulting irradiance distribution is the sum of every single one (Figure 9). Depending on the distance, the irradiance in the intermediate area between the two LEDs is enhanced, reduced, or constant. This dependence of the irradiance distribution from the LED distance is shown in Figure 9 for three different distances *d*. Using an array of *n*×*m* LEDs with *n* > 2 and *m*≥1, the overlapping effect is amplified. To achieve a uniform distribution, the right LED arrangement is crucial. The LED distances for a uniform irradiance depend on (i) the number of LEDs, (ii) the viewing angle θ_1/2_, and (iii) the working distance *z*. As a rule of thumb, for LEDs with a wide viewing angle, the distances of the inner LEDs should be wider than the distances of the outer ones.

However, a uniform irradiance comes with a price. Due to the positioning scheme, the average irradiance decreased by 10% compared with the equidistant arrangement. Simulations of different equidistant LED positions have shown that the resulting average irradiance increased by reducing the spacing between the individual LED, yet the uniformity decreased. Depending on the purpose of the irradiation system, a tradeoff between irradiance and homogeneity is necessary. For this work, a uniform light distribution was essential to accomplish comparable irradiation conditions within the 96-well plate.

Optical characterization measurements shown in Table 1 indicate that the actual peak wavelengths are within the specifications from the datasheets for all but the red LEDs. With a nominal wavelength range from λ = 624 to λ = 634 nm given by the manufacturer and an actual peak wavelength of λ = 640 nm, a divergence was observed. This deviation may result from a different characterization method in the datasheet. The LED manufacturer refers to a dominant wavelength, which takes the relative spectral sensitivity of human visual perception of brightness (luminosity function) into account (Lumileds Holding, 2019). The peak wavelength in this work refers to an absolute spectral measurement without considering the luminosity function of the human eye.

Irradiation measurements at the sample distance revealed a variation in the achieved intensities from a maximum irradiance of *E*_*m*_ = 13 ± 1.0 mW/cm^2^ for violet LEDs and a minimum irradiance of *E*_*m*_ = 1.1 ± 0.08 mW/cm^2^ for amber LEDs. These fluctuations can be explained by different designs and composition of each single-color LED. To achieve different emission wavelengths, different semiconductor combinations with different dopings are used in addition to various packaging layouts (Schubert, 2006). These intrinsic variations result in different irradiances.

### Establishment and Validation of a Screening Photoantimicrobial Assay

The EUCAST microdilution assay – being launched to allow a better inter-laboratory reproducibility – inspired the established photoantimicrobial HTS. Generally, while antimicrobial assays heavily depend on the testing conditions, one aim of EUCAST is to boost the development of new antimicrobials by the enablement of inter-laboratory comparisons. Specifically, photoantimicrobials are part of a promising treatment alternative, the so-called photoantimicrobial chemotherapy (PACT) or antimicrobial photodynamic inhibition (aPDI) (Wainwright, 2009). While the therapy depends on a completely different mechanism (i.e., ROS production due to the interplay of light and a photosensitizer) compared with well-established antibiotics, it is active against multi-resistant pathogens, and the risk of resistance development is relatively low (Maisch, 2015). Nevertheless, despite these attractive aspects, wide acceptance of PACT is lacking. One limitation is the restricted number of PSs in the pipeline of next-generation antibiotics. Next to others (Wainwright, 2009), the limited throughput of established assays is a bottle neck: Often, one PS-candidate and one concentration are irradiated by the time, leading to exorbitantly time-consuming experiments. On the other hand, testing multiple parameters (concentrations, microorganism, or drug-candidates) on one plate lacked comparability due to an uneven light distribution (Ogonowska et al., 2019). Thus, the limited throughput impeded the study of extensive libraries of PS candidates and, hence, the classical lead-to-hit approach of medicinal chemistry.

The EUCAST protocol tests an antibiotic (AB) usually with 10 concentrations and defines the MIC by the value, which is the lowest concentration inhibiting the growth completely as determined by the lack of turbidity. To test photoantimicrobials, a blank measurement of each concentration is necessary to avoid false-negative read-outs during the OD measurement. Furthermore, a triplicate of each concentration is needed to account for the biological variability. This led us to the (in Supplementary Figure 1) displayed, pipetting scheme, which allows testing the effect of two PSs against one microorganism. While for classic EUCAST susceptibility assays, only these variables (i.e., tested microorganism and concentration range of the AB) are crucial, the number of variables exceeds in the photoantimicrobial assay: In addition, the (i) preincubation time, (ii) irradiation time, and (iii) light doses, as well as (iv) light power and (v) the irradiation wavelength itself are of interest.

The established scheme (Figure 2) and the workability were tested with the known PSs curcumin, rose bengal (RB), methylene blue (MB), phenalenone (PN), and a *Hypericum* extract (HP). The irradiation wavelength changed according to the absorbance pattern of the PS (Figure 5). The light dose was set to *H* = 30 J/cm^2^, which equated to the utilized dose from several published studies (Cieplik et al., 2016; de Annunzio et al., 2018; Merigo et al., 2019) and, furthermore, was shown to be non-toxic against the tested microorganisms alone (Figure 4). While this is *per se* not as important as for photocytotoxicity studies (Wainwright, 2009), we chose this dose to truly see the light effect of the PSs. PhotoMIC values were generated for this variety of PSs against the pathogenic microorganisms *S. aureus*, *C. albicans*, and *E. coli* utilizing the LED modules with an irradiation wavelength of λ_irr_ = 428, 478, 523, 598, and 640 nm. To the best knowledge of the authors, Table 2 represents the first comprehensive overview of the PhotoMIC values of common PSs. Although literature values cannot be easily compared due to the discussed – not yet standardized parameters (Haukvik et al., 2009) – our obtained values correspond with the reported ones (see Supplementary Part, Chapter 2.2 for the full discussion). An international agreement on standard values for the additional irradiation parameters would be helpful in the process of hit-lead optimization.

The Gram-negative bacteria *E. coli* was resistant against the tested lipophilic and neutral PSs especially during the irradiation with yellow and red lights (Table 2). This is well-known (see discussion SI Chapter 2.2) and reasoned by their negatively charged membrane (Minnock et al., 2000; Bresolí-Obach et al., 2018; Galstyan et al., 2018).

To allow a screening of biological sources such as plant extracts or fungal extracts in the frame of bio-activity-guided isolation, the pipetting scheme displayed in Figure 2 was established. Due to the even light distribution, up to three concentrations of seven extracts can be screened against one pathogenic microorganism in biological triplicates. By a slight modification of the testing logic on the other hand, a fast determination of PhotoMIC against a broader variety of microorganisms in analogy to the EUCAST scheme is possible (i.e., up to seven microorganisms against one PSs, no triplicates).

### Utilization of the Screening Assay Yielded a Promising Hit

As a sample set, extracts of six different *Cortinarius* species (Table 3 and Supplementary Table 1), were investigated. The results of the antimicrobial assay (dark conditions) were in line with the results of Tiralongo and colleagues (Beattie et al., 2010). They investigated 117 different Australian *Cortinarius* species and could show that two-thirds of the species held an EC_50_ between *c* = 20 and *c* = 200 μg/ml against the Gram-positive bacterium *S. aureus*. In the present study, we determined MICs (instead of EC_50_), and were, under light exclusion, not able to see full inhibition of microbial growth with extract concentrations up to *c* = 75 μg/ml.

**FIGURE 10:**
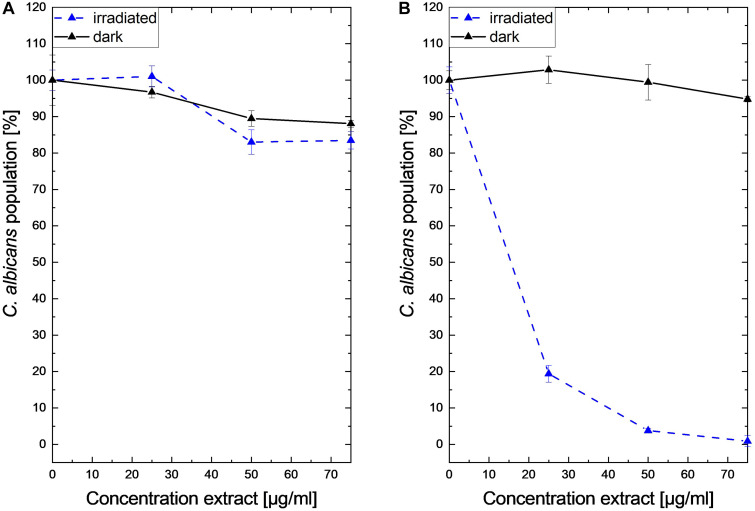
Dose–response curves of *C. albicans* treated with a *Cortinarius xanthophyllus* extract in the dark (black solid plot) and under blue light irradiation (blue dashed plots, λ = 478 nm, H = 30 J/cm^2^). On the left side **(A)**, the results with a preincubation time of *t* = 10 min are shown. The right graph **(B)** shows the growth inhibition after a preincubation time of *t* = 60 min.

As shown in Figure 6, the addition of blue light amplified the antimicrobial activity of the intensely colored *Cortinarius* extract by more than 10-fold: The extract of *C. xanthophyllus* was characterized by a PhotoMIC of *c* = 7.5 μg/ml and, thus, was even more effective than the established PS phenalenone (MIC = 25 μg/ml). In addition, the extract showed promising activity against *C. albicans* (Figure 6). Interestingly, this activity seemed to be uptake dependent, as a preincubation time of only 10 min (instead of PI = 60 min) showed no effect (Figure 10). Mycochemical analysis of the extract implicated four potential photoactive compounds (Supplementary Figure 12). These pigments were tentatively annotated as rufoolivacin A (Peak 2), rufoolivacin C (Peak 3), parietin (Peak 4), and as an anhydro-phlegmacin-like compound (Peak 5) (see also Supplementary Table 3). Analysis of the HPLC-DAD chromatogram recorded at λ = 478 nm indicated that Peak 4 and Peak 5 absorb most of the incoming light and, thus, might be responsible for the observed photoantimicrobial action. Parietin, usually isolated from the lichen *Xanthoria parietina*, is known for its photoactive properties against cancer cells (Mugas et al., 2021) and against *S. aureus* (Comini et al., 2017). The chemical structure of Peak 5 is not assured yet and is hampered by the limited availability of fungal material due to the rare occurrence of *C. xanthophyllus.* This Mediterranean species is listed on the red-list and, thus, is endangered (Tingstad et al., 2017). Nevertheless, applying modern phytochemical techniques (e.g., LC-SPE-NMR, FBMN-assisted isolation) might help to reveal its chemical space and is part of future work.

## Conclusion

The development of a uniform emitting LED panel was presented, allowing the homogeneous irradiation of a complete 96-well plate. As a consequence, a convenient HTS assay to determine photo-activated minimal inhibitory concentrations (PhotoMIC) of pure compounds and extracts was established based on the EUCAST guideline. The light tolerance of the utilized model organisms (i.e., *C. albicans*, *E. coli*, and *S. aureus*) was tested and revealed that all microorganisms can cope with a light dose of *H* = 9.3 J/cm^2^ or even *H* = 30 J/cm^2^ of every tested wavelength (i.e., up to 9.3 J/cm^2^ for λ = 598 nm, up to 30 J/cm^2^ for λ = 428, 478, 528, 640 nm). Standard photosensitizers were used to validate the assay and yielded the first comprehensive table accumulating a broad array of PhotoMIC values under different irradiation conditions and against different pathogenic MOs. Last, submitting a test sample set of fungal extracts generated from the colored fruiting bodies of *Cortinarius callisteus*, *C. rufo-olivaceus*, *C. traganus*, *C. trivialis*, *C. venetus*, and *C. xanthophyllus* showed that light can indeed be a co-factor amplifying the antimicrobial action of some natural products. The most intensely colored extract, i.e., the one of *C. xanthophyllus*, showed the most promising activity with a PhotoMIC = 7.5 μg/ml against *S. aureus*. The extract was also photoactive against *C. albicans*. Mycochemical analysis identified two peaks putatively responsible for the effect, one of them being the well-known natural PS parietin from the lichen *Xanthoria parietina* and the other one being a photochemically unexplored dimeric AQ.

## Data Availability Statement

The datasets presented in this study can be found in online repositories. The names of the repository/repositories and accession number(s) can be found below: https://www. ncbi.nlm.nih.gov/genbank/, MW880290; https://www.ncbi.nlm. nih.gov/genbank/, MW898452; https://www.ncbi.nlm.nih.gov/genbank/, MW880292; https://www.ncbi.nlm.nih.gov/genbank/, MW871552; https://www.ncbi.nlm.nih.gov/genbank/, MW898453; https://www.ncbi.nlm.nih.gov/genbank/, MW880291.

## Author Contributions

JF performed the antimicrobial assays and majority of the mycochemical analysis. FH performed the DMA assay. HS and RS designed the irradiation device. HS performed the instrumental characterization. DD and DA performed the pre-test of the AntiMic assay. PV contributed to the conception of the AntiMic assay. UP provided the biomaterial and phylogenetic input. BS designed the research, analyzed the mycochemical part, and wrote the manuscript with contributions of HS and JF. All authors contributed to the final version of the manuscript.

## Conflict of Interest

The authors declare that the research was conducted in the absence of any commercial or financial relationships that could be construed as a potential conflict of interest.

## Publisher’s Note

All claims expressed in this article are solely those of the authors and do not necessarily represent those of their affiliated organizations, or those of the publisher, the editors and the reviewers. Any product that may be evaluated in this article, or claim that may be made by its manufacturer, is not guaranteed or endorsed by the publisher.

## Footnotes

1
https://www.dsmz.de/

## References

[B1] Bajgar R. Pola M. Hosik J. Turjanica P. Cengery J. Kolarova H. (2020). New planar light source for the induction and monitoring of photodynamic processes in vitro. *J. Biol. Phys.* 46 121–131. 10.1007/s10867-020-09544-7 32170534 PMC7098404

[B2] Balouiri M. Sadiki M. Ibnsouda S. K. (2016). Methods for in vitro evaluating antimicrobial activity: a review. *J. Pharm. Anal.* 6 71–79. 10.1016/j.jpha.2015.11.005 29403965 PMC5762448

[B3] Basnet B. B. Liu L. Bao L. Liu H. (2017). Current and future perspective on antimicrobial and anti-parasitic activities of *Ganoderma* sp.: an update. *Mycology* 8 111–124. 10.1080/21501203.2017.1324529 30123634 PMC6059132

[B4] Beattie K. D. Rouf R. Gander L. May T. W. Ratkowsky D. Donner C. D. (2010). Antibacterial metabolites from Australian macrofungi from the genus *Cortinarius*. *Phytochemistry* 71 948–955. 10.1016/j.phytochem.2010.03.016 20392467

[B5] Benkova M. Soukup O. Marek J. (2020). Antimicrobial susceptibility testing: currently used methods and devices and the near future in clinical practice. *J. Appl. Microbiol.* 129 806–822. 10.1111/jam.14704 32418295

[B6] Berenbaum M. (1995). Phototoxicity of plant secondary metabolites: insect and mammalian perspectives. *Arch. Insect Biochem. Physiol.* 29 119–134. 10.1002/arch.940290204 7606040

[B7] Betts J. T. (1976). Solving the nonlinear least square problem: application of a general method. *J. Optim. Theory Appl.* 18 469–483. 10.1007/bf00932656

[B8] Bresolí-Obach R. Gispert I. Peña D. G. Boga S. Gulias Ó Agut M. (2018). Triphenylphosphonium cation: a valuable functional group for antimicrobial photodynamic therapy. *J. Biophotonics* 11:e201800054. 10.1002/jbio.201800054 29882394

[B9] Butler M. C. Itotia P. N. Sullivan J. M. (2010). A High-throughput biophotonics instrument to screen for novel ocular photosensitizing therapeutic agents. *Invest. Ophthalmol. Vis. Sci.* 51 2705–2720. 10.1167/iovs.08-2862 19834043 PMC2868480

[B10] Calin M. A. Parasca S. V. (2009). Light sources for photodynamic inactivation of bacteria. *Lasers Med. Sci.* 24 453–460. 10.1007/s10103-008-0588-5 18622686

[B11] Chen D. Zheng H. Huang Z. Lin H. Ke Z. Xie S. (2012). Light-emitting diode-based illumination system for in vitro photodynamic therapy. *Int. J. Photoenergy* 2012:920671.

[B12] Cieplik F. Pummer A. Leibl C. Regensburger J. Schmalz G. Buchalla W. (2016). Photodynamic inactivation of root canal bacteria by light activation through human dental hard and simulated surrounding tissue. *Front. Microbiol.* 7:929. 10.3389/fmicb.2016.00929 27379059 PMC4908107

[B13] Coleman T. F. Li Y. (1994). On the convergence of interior-reflective Newton methods for nonlinear minimization subject to bounds. *Math. Program.* 67 189–224. 10.1007/bf01582221

[B14] Coleman T. F. Li Y. (1996). An interior trust region approach for nonlinear minimization subject to bounds. *SIAM J. Opt.* 6 418–445. 10.1137/0806023

[B15] Comini L. R. Moran Vieyra F. E. Mignone R. A. Paez P. L. Laura Mugas M. Konigheim B. S. (2017). Parietin: an efficient photo-screening pigment in vivo with good photosensitizing and photodynamic antibacterial effects in vitro. *Photochem. Photobiol. Sci.* 16 201–210. 10.1039/c6pp00334f 27976779

[B16] de Annunzio S. R. De Freitas L. M. Blanco A. L. Da Costa M. M. Carmona-Vargas C. C. De Oliveira K. T. (2018). Susceptibility of Enterococcus faecalis and *Propionibacterium acnes* to antimicrobial photodynamic therapy. *J. Photochem. Photobiol. B Biol.* 178 545–550. 10.1016/j.jphotobiol.2017.11.035 29253813

[B17] Deveau A. Bonito G. Uehling J. Paoletti M. Becker M. Bindschedler S. (2018). Bacterial–fungal interactions: ecology, mechanisms and challenges. *FEMS Microbiol. Rev.* 42 335–352. 10.1093/femsre/fuy008 29471481

[B18] Dos Santos R. F. Campos B. S. Rego Filho F. Moraes J. O. Albuquerque A. L. I. Da Silva M. C. D. (2019). Photodynamic inactivation of S. aureus with a water-soluble curcumin salt and an application to cheese decontamination. *Photochem. Photobiol. Sci.* 18 2707–2716. 10.1039/c9pp00196d 31556891

[B19] Downum K. R. (1992). Light-activated plant defence. *New Phytologist* 122 401–420. 10.1111/j.1469-8137.1992.tb00068.x 33874213

[B20] Dresch P. D’Aguanno M. N. Rosam K. Grienke U. Rollinger J. M. Peintner U. (2015). Fungal strain matters: colony growth and bioactivity of the European medicinal polypores Fomes fomentarius, *Fomitopsis pinicola* and *Piptoporus betulinus*. *AMB Express* 5:4. 10.1186/s13568-014-0093-0 PMC4305089 25642401

[B21] Elsworth C. Gill M. Giménez A. M. Milanovic N. Raudies E. (1999). Pigments of fungi. Part 50.1 structure, biosynthesis and stereochemistry of new dimeric dihydroanthracenones of the phlegmacin type from *Cortinarius* sinapicolor Cleland. *J. Chem. Soc. Perkin Trans.* 1 119–126. 10.1039/a808340a

[B22] Espinoza C. Trigos Á Medina M. E. (2016). Theoretical study on the photosensitizer mechanism of phenalenone in aqueous and lipid media. *J. Phys. Chem. A* 120 6103–6110. 10.1021/acs.jpca.6b03615 27428932

[B23] Fiala J. Schöbel H. Vrabl P. Dietrich D. Hammerle F. Altmann D. J. (2021). A new high-throughput-screening-assay for photoantimicrobials based on EUCAST revealed photoantimicrobials in Cortinariaceae. *bioRxiv*[Preprint] 2021.2004.2002.438202, 10.3389/fmicb.2021.703544 PMC8375034 34421861

[B24] Flors C. Nonell S. (2006). Light and singlet oxygen in plant defense against pathogens: phototoxic phenalenone phytoalexins. *Acc. Chem. Res.* 39 293–300. 10.1021/ar0402863 16700528

[B25] Frey-Klett P. Burlinson P. Deveau A. Barret M. Tarkka M. Sarniguet A. (2011). Bacterial-fungal interactions: hyphens between agricultural, clinical, environmental, and food microbiologists. *Microbiol. Mol. Biol. Rev.* 75 583–609. 10.1128/mmbr.00020-11 22126995 PMC3232736

[B26] Galstyan A. Putze J. Dobrindt U. (2018). Gaining Access to bacteria through (reversible) control of lipophilicity. *Chem. Eur. J.* 24 1178–1186. 10.1002/chem.201704562 29117462

[B27] Gao J.-M. Qin J.-C. Pescitelli G. Di Pietro S. Ma Y.-T. Zhang A.-L. (2010). Structure and absolute configuration of toxic polyketide pigments from the fruiting bodies of the fungus *Cortinarius* rufo-olivaceus. *Org. Biomol. Chem.* 8 3543–3551. 10.1039/c002773a 20532365

[B28] Gill M. Steglich W. (1987). Pigments of fungi (macromycetes). *Fortschr. Chem. Org. Naturst.* 51 1–317. 3315906 10.1007/978-3-7091-6971-1_1

[B29] Hammerle F. Bingger I. Pannwitz A. Magnutzki R. Gstir A. Rutz J.-L. (2021). Targeted isolation of photoactive pigments from mushrooms yielded a highly potent new photosensitizer: 7,7′-Biphyscion. *ChemRXiv*. 10.1016/j.phymed.2019.152985 35064132 PMC8782903

[B30] Haukvik T. Bruzell E. Kristensen S. Tønnesen H. H. (2009). Photokilling of bacteria by curcumin in different aqueous preparations. Studies on curcumin and curcuminoids XXXVII. *Pharmazie* 64 666–673. 19947170

[B31] Hernández Quintanar L. F. López Silva F. Y. Fabila Bustos D. A. Serrano Navarro J. de La Rosa Vázquez J. M. (2016). In vitro photoirradiation system for simultaneous irradiation with different light doses at a fixed temperature. *Photomed. Laser Surg.* 34 108–115. 10.1089/pho.2015.4030 26890993

[B32] Hofbauer C. (1983). *Chemotaxonomische Untersuchungen in der Untergattung Phlegmacium.* Ph.D Dissertation. Innsbruck: University of Innsbruck.

[B33] Hopkins S. L. Siewert B. Askes S. H. C. Veldhuizen P. Zwier R. Heger M. (2016). An in vitro cell irradiation protocol for testing photopharmaceuticals and the effect of blue, green, and red light on human cancer cell lines. *Photochem. Photobiol. Sci.* 15 644–653. 10.1039/c5pp00424a 27098927 PMC5044800

[B34] Hudson J. B. Towers G. H. (1991). Therapeutic potential of plant photosensitizers. *Pharmacol. Ther.* 49 181–222. 10.1016/0163-7258(91)90055-q 2052625

[B35] Hyde K. D. Xu J. Rapior S. Jeewon R. Lumyong S. Niego A. G. T. (2019). The amazing potential of fungi: 50 ways we can exploit fungi industrially. *Fungal Divers.* 97 1–136.

[B36] Katz S. Backeris P. Merck C. Suprun M. D’souza S. Bishop D. F. (2018). Design and validation of an open-source modular microplate photoirradiation system for high-throughput photobiology experiments. *PLoS One* 13:e0203597. 10.1371/journal.pone.0203597 30289930 PMC6173374

[B37] Künzler M. (2018). How fungi defend themselves against microbial competitors and animal predators. *PLoS Pathog.* 14:e1007184. 10.1371/journal.ppat.1007184 30188951 PMC6126850

[B38] Lachowicz J. I. Dalla Torre G. Cappai R. Randaccio E. Nurchi V. M. Bachor R. (2020). Metal self-assembly mimosine peptides with enhanced antimicrobial activity: towards a new generation of multitasking chelating agents. *Dalton Trans.* 49 2862–2879. 10.1039/c9dt04545g 32067003

[B39] Lee I. H. Cho Y. Lehrer R. I. (1997). Effects of pH and salinity on the antimicrobial properties of clavanins. *Infect. Immun.* 65 2898–2903. 10.1128/iai.65.7.2898-2903.1997 9199465 PMC175407

[B40] Lewis K. (2020). The Science of antibiotic discovery. *Cell* 181 29–45. 10.1016/j.cell.2020.02.056 32197064

[B41] Lumileds Holding B. V. (2019). *DS198 LUXEON CZ Color Line Product Datasheet [Online].* Available online at: https://www.lumileds.com/products/color- leds/luxeon-cz-color-line/ (accessed 01.02.2021 2021)

[B42] Maisch T. (2015). Resistance in antimicrobial photodynamic inactivation of bacteria. *Photochem. Photobiol. Sci.* 14 1518–1526. 10.1039/c5pp00037h 26098395

[B43] Merigo E. Conti S. Ciociola T. Manfredi M. Vescovi P. Fornaini C. (2019). Antimicrobial photodynamic therapy protocols on *Streptococcus mutans* with different combinations of wavelengths and photosensitizing dyes. *Bioengineering (Basel)* 6:42. 10.3390/bioengineering6020042 31083438 PMC6631272

[B44] Mathworks I. (2019). *“MATLAB R2019b”. 9.7.0.1296695 (R2019b) Update*, 4th Edn. Natick, MA.

[B45] Microbiology and Diseases (2003). Determination of minimum inhibitory concentrations (MICs) of antibacterial agents by broth dilution. *Clin. Microbiol. Infect.* 9 ix–xv. 10.1046/j.1469-0691.2000.00142.x 11168187

[B46] Minnock A. Vernon D. I. Schofield J. Griffiths J. Parish J. H. Brown S. B. (2000). Mechanism of uptake of a cationic water-soluble pyridinium zinc phthalocyanine across the outer membrane of *Escherichia coli*. *Antimicrob. Agents Chemother.* 44 522–527. 10.1128/aac.44.3.522-527.2000 10681312 PMC89720

[B47] Moreno I. Avendaño-Alejo M. Tzonchev R. I. (2006). Designing light-emitting diode arrays for uniform near-field irradiance. *Appl. Opt.* 45 2265–2272. 10.1364/ao.45.002265 16607994

[B48] Morici P. Battisti A. Tortora G. Menciassi A. Checcucci G. Ghetti F. (2020). The in vitro photoinactivation of *Helicobacter* pylori by a novel LED-based device. *Front. Microbiol.* 11:283. 10.3389/fmicb.2020.00283 32153551 PMC7047934

[B49] Moser M. (1972). Die Gattung Dermocybe (Fr.) Wünsche (Die Hautköpfe). *Schw.Zeitschrift Pilzkunde Sondernummer* 83 153–167.

[B50] Mugas M. L. Calvo G. Marioni J. Céspedes M. Martinez F. Sáenz D. (2021). Photodynamic therapy of tumour cells mediated by the natural anthraquinone parietin and blue light. *J. Photochem. Photobiol. B Biol.* 214 112089. 10.1016/j.jphotobiol.2020.112089 33271387

[B51] Nielsen H. K. Garcia J. Væth M. Schlafer S. (2015). Comparison of Riboflavin and toluidine blue O as photosensitizers for photoactivated disinfection on endodontic and periodontal pathogens in vitro. *PLoS One* 10:e0140720. 10.1371/journal.pone.0140720 26469348 PMC4607437

[B52] Ogonowska P. Woźniak A. Pierański M. Wasylew T. Kwiek P. Brasel M. (2019). Application and characterization of light-emitting diodes for photodynamic inactivation of bacteria. *Light. Res. Technol.* 51 612–624. 10.1177/1477153518781478

[B53] Pieslinger A. Plaetzer K. Oberdanner C. B. Berlanda J. Mair H. Krammer B. (2006). Characterization of a simple and homogeneous irradiation device based on light-emitting diodes: a possible low-cost supplement to conventional light sources for photodynamic treatment. *Med. Laser Appl.* 21 277–283. 10.1016/j.mla.2006.07.004

[B54] Reifegerste F. Lienig J. (2008). Modelling of the temperature and current dependence of LED spectra. *J. Light Vis. Environ.* 32 288–294. 10.2150/jlve.32.288

[B55] Rodriguez-Tudela J. L. Arendrup M. C. Barchiesi F. Bille J. Chryssanthou E. Cuenca-Estrella M. (2008). EUCAST definitive document EDef 7.1: method for the determination of broth dilution MICs of antifungal agents for fermentative yeasts: subcommittee on antifungal susceptibility testing (AFST) of the ESCMID european committee for antimicrobial susceptibility testing (EUCAST)^∗^. *Clin. Microbiol. Infect.* 14 398–405. 10.1111/j.1469-0691.2007.01935.x 18190574

[B56] Schmidt R. Tanielian C. Dunsbach R. Wolff C. (1994). Phenalenone, a universal reference compound for the determination of quantum yields of singlet oxygen O2(1Δg) sensitization. *J. Photochem. Photobiol. A Chem.* 79 11–17. 10.1016/1010-6030(93)03746-4

[B57] Schubert E. F. (2006). *Light-Emitting Diodes*, Second Edn. Cambridge: Cambridge University Press.

[B58] Siewert B. (2021). Does the chemistry of fungal pigments demand the existence of photoactivated defense strategies in basidiomycetes? *Photochem. Photobiol. Sci.* 20 475–488. 10.1007/s43630-021-00034-w 33738747

[B59] Siewert B. Stuppner H. (2019). The photoactivity of natural products – an overlooked potential of phytomedicines? *Phytomedicine* 60:152985. 10.1016/j.phymed.2019.152985 31257117

[B60] Siewert B. Vrabl P. Hammerle F. Bingger I. Stuppner H. (2019). A convenient workflow to spot photosensitizers revealed photo-activity in basidiomycetes. *RSC Adv.* 9 4545–4552. 10.1039/c8ra10181g 30931108 PMC6394893

[B61] Supronowicz R. Fryc I. (2019). “The LED spectral power distribution modelled by different functions – how spectral matching quality affected computed LED color parameters,” in *Proceedings of the 2019 Second Balkan Junior Conference on Lighting (Balkan Light Junior)*) (Plovdiv: IEEE), 1–4. 10.1117/1.oe.58.3.035105

[B62] Tingstad L. Gjerde I. Dahlberg A. Grytnes J. A. (2017). The influence of spatial scales on Red List composition: forest species in Fennoscandia. *Glob. Ecol. Conserv.* 11 247–297. 10.1016/j.gecco.2017.07.005

[B63] Wainwright M. (2009). Photoantimicrobials—so what’s stopping us? *Photodiagnosis Photodyn. Ther.* 6 167–169. 10.1016/j.pdpdt.2009.10.007 19932448

[B64] Weinstein M. P. Lewis J. S. (2020). The clinical and laboratory standards institute subcommittee on antimicrobial susceptibility testing: background, organization, functions, and processes. *J. Clin. Microbiol.* 58:e01864-19. 10.1128/JCM.01864-19 PMC7041576 31915289

[B65] Wiegand C. Abel M. Ruth P. Elsner P. Hipler U. C. (2015). pH Influence on antibacterial efficacy of common antiseptic substances. *Skin Pharmacol. Physiol.* 28 147–158. 10.1159/000367632 25614073

[B66] Wiegand I. Hilpert K. Hancock R. E. W. (2008). Agar and broth dilution methods to determine the minimal inhibitory concentration (MIC) of antimicrobial substances. *Nat. Protoc.* 3 163–175. 10.1038/nprot.2007.521 18274517

[B67] Wood D. (1994). *Optoelectronic Semiconductor Devices.* Hoboken, NJ: Prentice Hall.

[B68] Wozniak A. Grinholc M. (2018). Combined antimicrobial activity of photodynamic inactivation and antimicrobials–state of the art. *Front. Microbiol.* 9:930. 10.3389/fmicb.2018.00930 29867839 PMC5952179

[B69] Zhang A. L. Qin J. C. Bai M.-S. Gao J. M. Zhang Y. M. Yang S. X. (2009). Rufoolivacin B, a novel polyketide pigment from the fruiting bodies of the fungus *Cortinarius* rufo-olivaceus (basidiomycetes). *Chin. Chem. Lett.* 20 1324–1326. 10.1016/j.cclet.2009.05.021

