# Supplementary material for: A New High-Throughput-Screening-Assay for Photoantimicrobials Based on EUCAST Revealed Unknown Photoantimicrobials in Cortinariaceae
Source: Front Microbiol. 2021 Aug 5;12:703544. doi: 10.3389/fmicb.2021.703544 (PMC8375034; doi:10.3389/fmicb.2021.703544)
Supplement: Supplementary file 1 [file Data_Sheet_1.pdf]

Supplementary Material

1 Fungal Material

Table S1. *Cortinarius* collections used in this study with respective voucher numbers and collection data.

	Voucher/ GenBank	leg. et det.	Origin
C. traganus	IBF20180231 / MW880290	14.10.2017	Pian di Carniglia, Bedonia, Italy
C. rufoolivaceus	IBF20190113 / MW898452	20.10.2019	Oasi Ghirardi, Bedonia, Italy
C. venetus (Fr.) Fr.	IBF20180223 / MW880292	15.10.2018	Orto Botanico Forestale dell'Abetone, Italy
C. callisteus	IBF20190145 / MW871552	22.10.2019	Stabielle, Bedonia, Italy
C. xanthophyllus	IBF20050337 / MW898453	19.10.2019	Val di Taro, Stabielle, Borgotar, Italy
C. trivialis	IBF20170586 / MW880291	17.10.2017	Massa Carrara Passo, Parma, Bedonia, Italy

2 Photoantimicrobial Assays

2.1 Pipetting Scheme for photoantimicrobials

In Figure S1 a pipetting scheme for photoantimicrobials is presented.

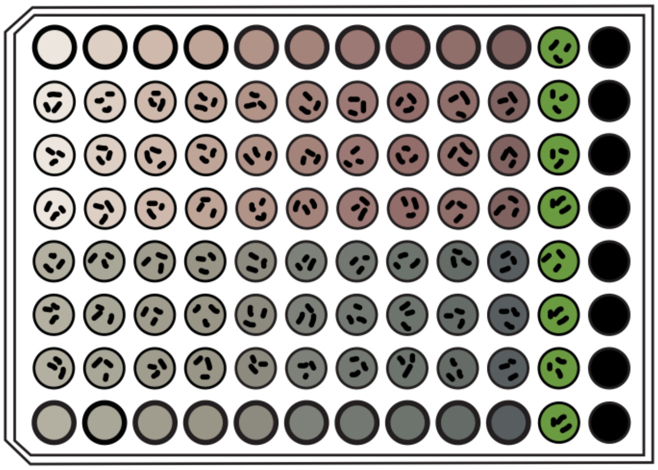

Figure S1. Pipetting scheme for two antimicrobials (Compound A = brown and B = grey) tested against one microorganism in ten different, from left to right rising, concentrations. Bold strokes imply the blank of each tested concentration. GC = Growth control = green vials, SC = Sterility control = full black vials.

2.2 (Photo)antimicrobial action of positive controls

2.2.1 Curcumin

Our experimental PhotoMIC for curcumin (c = 4 µg/mL, 10.9 µM, *H* = 30 J/cm², λ = 428 ± 15 nm, 60 min PI, Figure S2) against *Candida albicans* concurred with the findings of previous studies (Carmello et al., 2015;2017), where a minimum fungicidal concentration (MFC) of c = 20 µg/mL (54.3 µM) was reported. They utilized a similar light dose (*H* = 37.5 J/cm^2) and a comparable light source (λ = 455 -15/+5 nm) but a three times shorter PI (t = 20 min). The potential influence of PI for PACT against *C. albicans* can also be seen in the results of *C. xanthophyllus* against *C. albicans* (Figure S14). Although MFCs are higher than MICs, the results are moderately comparable. In line with previously reported data (Dovigo et al., 2011), irradiation with λ = 478 ± 28 nm light and a fluence of 9.3 J/cm^2 increases the MIC to c = 30 µg/mL (81.4 µM) (Table 2, Figure S4).

The reported photoantimicrobial actions of curcumin against the gram-negative bacteria *Escherichia coli* are quite controversial: Colleagues found a 5.94 log CFU/ml reduction (λ = 462 nm, *H* = 13 J/cm^2, growth condition = 75 mm petri dish) with a concentration of c = 7.37 µg/mL (20.0 µM) (Bhavya and Umesh Hebbar, 2019). The same group also reported that a PI up to t = 60 min did not show any significant difference on the results in their experimental setup.
Another group reported a reduction of up to 3 log CFU/mL at a concentration of c = 5 µg/mL (13.57 µM) and a smaller reduction of 1.8 log CFU/mL with a concentration of c = 10 µg/mL (27.15 µM) (Medium = citrate buffer (c = 100 µM), growth conditions = 12-well flat bottom polystyrene plate, light source = UV-A lamps, λ = 320–400 nm, PI = 5 min) (de Oliveira et al., 2018). In another report, reductions of just 1.29 and 2.65 log CFU/ml were found when *E. coli* was treated with curcumin (c = 27.63 µg/mL, 75 µM) under comparatively strong irradiation conditions (λ = 470 nm, *H* = 139 and 278 J/cm^2, respectively, PI = 10 min) (Penha et al., 2017). Our experimental PhotoMIC (*H* = 30 J/cm², λ = 428 ± 15 nm, PI = 10 min, Figure S1) for curcumin against *E. coli* equalled c = 40 µg/mL (108.6 µM).
Reasons for these controversial results might be the different irradiation setups, the preincubation time, the method (i.e., microdilution assay *vs* colony forming assay) itself, or the different susceptibilities of different strains.

Against *Staphylococcus aureus* a MIC of c = 4 µg/mL (10.86 µM) curcumin (λ = 430 nm, *H* = 9.3 J/cm², PI = 10 min) was found by us (Table 2, Figure S1). With a concentration of c = 2 µg/mL (5.43 µM) a 50% inhibition of growth was shown. Previous studies reported a 2 log CFU/ml reduction at c = 2.5 µM (0.92 µg/mL) with a smaller light dose and a longer PI (λ = 470 nm, *H* = 3 J/cm^2, PI = 60 min) (Jiang et al., 2014).

2.2.2 Phenalenone

Phenalenone derivatives are known to be promising natural PSs (Flors and Nonell, 2006). The lipophilic backbone structure itself, however, showed in previous experiments against *S. aureus*, *E. faecalis*, and *E. coli* –despite extremely promising photochemical properties (Φ_Δ = 1)– no photoactivity at low concentrations and low light doses (c = 1.80 µg/mL, 10.0 µM, λ = 420 ± 10 nm, *H* = 1.2 J/cm^2, PI = 10 min) (Bresolí-Obach et al., 2018). Applying an 8-times higher light dose, however, we discovered a weak photoantimicrobial effect against *S. aureus* and *E. coli*. In detail, PhotoMICs of c = 25 µg/mL (138.7 µM) and c = 75 µg/mL (416.2 µM) were found, respectively (Figure S 3).

In line with previous reports (c = 1.13 µg/mL – 90.10 µg/mL (6.25 – 500 µM), λ = 380 - 500 nm, H = 12 J/cm^2, PI = 240 min), (Bauer, 2016)), we found no photoactivity of phenalenone against *C. albicans* with a similar light dose (H = 9.3 J/cm^2) and concentrations up to c = 75 µg/ml (400 µM).

2.2.3 Rose bengal

With a concentration of c = 48.69 µg/mL (50.0 µM) rose bengal (RB), a 7.73 log CFU/ml reduction for *E. coli* using a light dose of H = 94.74 J/cm^2 was previously reported (λ = 460 nm (no deviation reported), PI = 5 min) (Rossoni et al., 2010). Our PhotoMIC was determined as c = 150 µg/mL (154.1 µM) using a third of the previous applied light dose (H = 30 J/cm^2, PI = 10 min). For an even lower light dose (H = 4.53 J/cm^2), a MIC below a concentration of c = 194.74 µg/mL (200.0 µM) was not reported against *E. coli* (λ = 400 – 700 nm, H = 4.53 J/cm^2, no PI reported) (Nisnevitch et al., 2010). Thus, the photoantimicrobial effect of RB is clearly concentration and light-dose dependend.

For different strains of *S. aureus,* PhotoMICs between c = 0.625 – 2.50 µg/mL, (0.642 – 2.568 µM, λ = 400 – 700 nm, H = 2.265 J/cm^2, no PI reported) were found for RB (Ilizirov et al., 2018). Similar concentrations were determined by another group reporting MIC values of c = 2.92 µg/mL (3 µM) with comparable irradiation settings (λ = 400 – 700 nm, H = 4.53 J/cm^2, no PI reported) (Nisnevitch et al., 2010). Employing our PhotoMIC assay we determined a slightly higher MIC of c = 4 µg/mL (4.1 µM) using a higher light dose (λ = 523 ± 33 nm, H = 30 J/cm^2, PI = 10 min, Figure S5). The fact that despite an higher light dose an higher PhotoMIC was found can not be entirely explained. It might be that the PI –being not reported in the previous studies– has a crucial effect on the photoantimicrobial action of RB against the gram positive germ.

2.2.4 Hypericum perforatum Extract

With the easily available ethanolic *Hypericum perforatum* extract, we were able to evaluate our assay with the photoactive natural product hypericin. As recently presented, hypericin is highly active against *S. aureus* (reduction of 7 log CFU/mL at a concentration of c = 0.50 µg/mL (1 µM), λ = 515 ± 40 nm, H = 9.6 J/cm^2, PI = 30 min) (Delcanale et al., 2020).
The antibacterial activity was confirmed by us (Figure S4 and Figure S5) and in addition we were able to observe the photodynamic inhibition of growth against *C. albicans* at different wavelengths of irradiation (Table 2). It is difficult to compare the MIC values of the pure substance Hypericin with the whole ethanolic extract from *Hypericum perforatum* (i.e., c = 150 µg/ml, λ = 598 ± 16 nm, H = 9.6 J/cm^2, PI = 10 min). Nevertheless, the extract proofed sufficient for finding MICs against *S. aureus* and *C. albicans*, showing photoantimicrobial activity in accord with previous studies using pure Hypericin (Alam et al., 2019).

2.2.5 Methylene blue

For methylene blue (MB) we found a PhotoMIC of c = 2.5 µg/mL (7.8 µM) against *C. albicans* (λ = 640 nm, H = 30 J/cm^2, PI = 60 min, Figure S6). This value fits in between the diverging literature results: Previous studies found a maximal reduction of MO growth with c = 1 µg/mL (3.1 µM) (λ = 630 nm (no deviation and light dose reported), PI = 30 min) (Pasyechnikova et al., 2009). Colleagues found an inhibitory concentration of c = 0.01 µg/mL (0.03 µM) for their setup (λ = 660 nm, H = 19.2 J/cm^2, PI = 10 min) (Hosseini et al., 2016). Higher PhotoMICs are reported by another group, who used a concentration of c = 6.40 µg/mL (20.0 µM) of MB (λ = 600 - 650 nm, H = 60 J/cm^2, PI = 30 min) to achieve a growth inhibition of 99% for *C. albicans* (Torres-Hurtado et al., 2019). Alternatively, 20 µg/mL (62.53 µM) are reported as the optimal concentration for a different setup (λ = 640 ± 12 nm, H = 4.68 J/cm^2). Different PIs (1 - 20 min) did not influence the results (da Collina et

al., 2018). Another group reported a concentration of c = 50 µg/mL (156.32 µM) as the optimal MB concentration to inhibit 80 - 90% of the *C. albicans* growth after irradiation (λ = 684 nm (no deviation reported), *H* = 28 J/cm^2, PI = 5 min) (Carvalho et al., 2009).

Against *E. coli* a concentration of c = 11.3 µg/mL (35.2 µM) (λ = 665 -65/+35 nm, *H* = 4 J/cm^2, no PI reported) leading to 93.7% inhibition of growth was reported (Peloi et al., 2008). In older studies total cell death was induced with a three-times higher concentration (c = 31.4 µg/mL, 100 µM) of MB (*H* = 6.3 J/cm^2, no further details reported) (Wainwright et al., 1997). Our findings of a PhotoMIC for MB against *E. coli* are to be found in between the reported results, but we decided not to use MB as a PC against *E. coli* because the light enhanced effect was too small (i.e., light toxicity (c = 20 µg/mL (63.7 µM), λ = 640 ± 18 nm, *H* = 30 J/cm^2, PI = 10 min) *vs* dark toxicity (c = 30 µg/mL (95.5 µM)).

The dark toxicity of MB against *E. coli* was recently discussed, raising the question of resistance development in certain strains (Gunics et al., 2000; Thesnaar et al., 2021). Overlapping light and dark toxicity for MB against several MOs is known, such as *Enterococcus faecalis* and *E. faecium* (Wainwright et al., 1999) or *S. aureus* (Wainwright et al., 1998) (Wainwright and Crossley, 2002). For antimicrobial activity of MB against *S. aureus* in the dark the MIC was reported with a concentration of c = 16 µg/mL (50.0 µM) (Thesnaar et al., 2021). Future research should investigate if a lower concentration of MB with a higher light dose and an experimental setup with stricter exclusion of surrounding light extends the therapeutical window of MB.

According to the differences found between the MIC values for the various PS and wavelengths, species and strains, a standardized procedure for PhotoMICs similar to the EUCAST or CLSI protocols will significantly improve the comparability.

2.3 Dose-response curves of tested MOs against PCs with different wavelengths of light irradiation

2.3.1 Dose-response curves of irradiation with blue-light

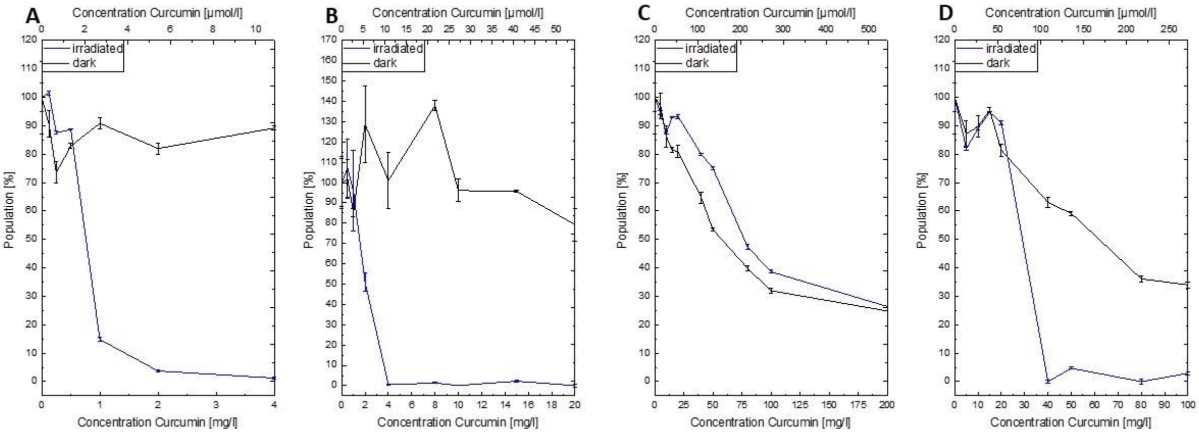

Figure S2. (Photo)antimicrobial action of curcumin against A) *C. albicans* (λ = 430 nm, *H* = 30 J/cm^2, PI = 60 min), B) *S. aureus* (λ = 430 nm, *H* = 9.3 J/cm^2, PI = 10 min), C) *Escherichia coli* with a lower light dose (λ = 430 nm, *H* = 9.3 J/cm^2, PI = 10 min,), and D) *E. coli* at a higher light dose (λ = 430 nm, *H* = 30 J/cm^2, PI = 10 min).

2.3.2 Dose-response curves of irradiation with purple-light

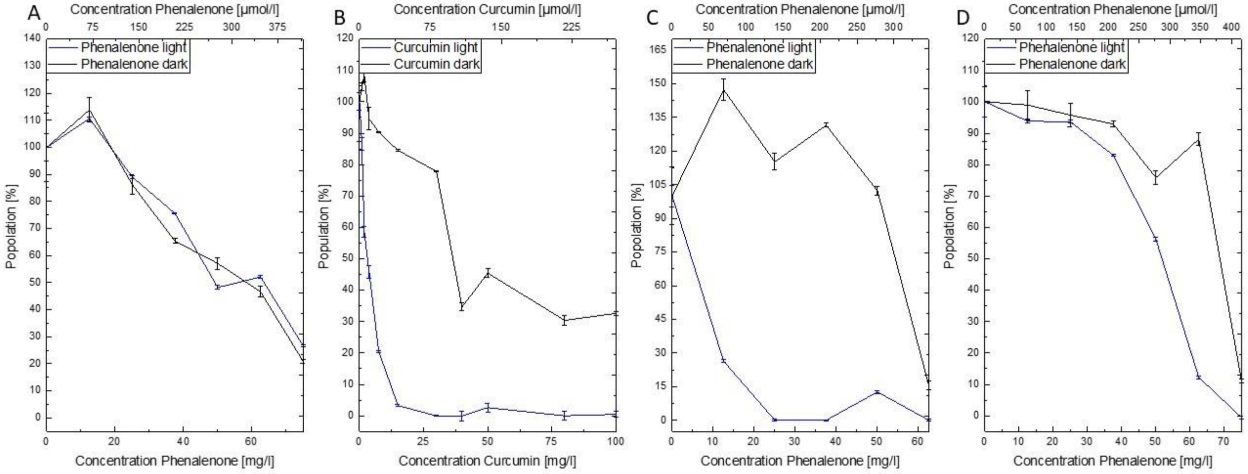

Figure S 3 (Photo)antimicrobial action of phenalenone (A, C, D) and curcumin (B) used as PC against A) *C. albicans* (λ = 478 nm, *H* = 9.3 J/cm^2, PI = 10 min), B) *S. aureus* (λ = 478 nm, *H* = 9.3 J/cm^2, PI = 10 min), C) Phenalenone against *S. aureus* (λ = 478 nm, H = 9.3 J/cm^2, PI = 10 min), D) Phenalenone against *E. coli* (λ = 478 nm, H = 9.3 J/cm^2, PI = 10 min).

2.3.3 Dose-response curves of irradiation with green-light

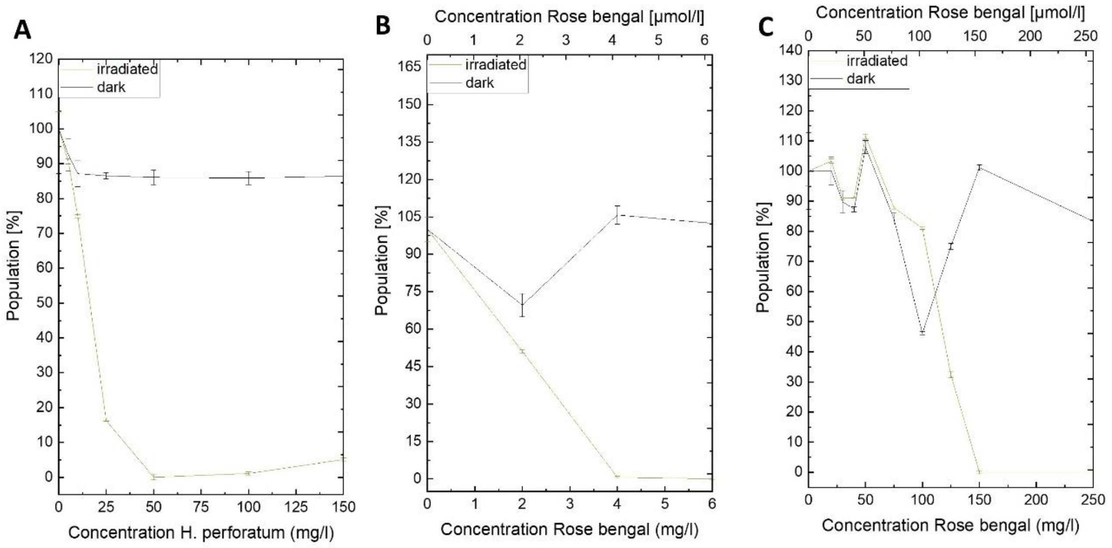

Figure S4. Dose-response curves recorded for A) HP against *C. albicans* (λ = 523 nm, H = 30 J/cm^2, PI = 10 min), B) RB against *E. coli* (λ = 523 nm, H = 30 J/cm^2, PI = 10 min), C) RB against *S. aureus* (λ = 523 nm, H = 30 J/cm^2, PI = 10 min).

2.4 Dose-response curves of irradiation with orange-light

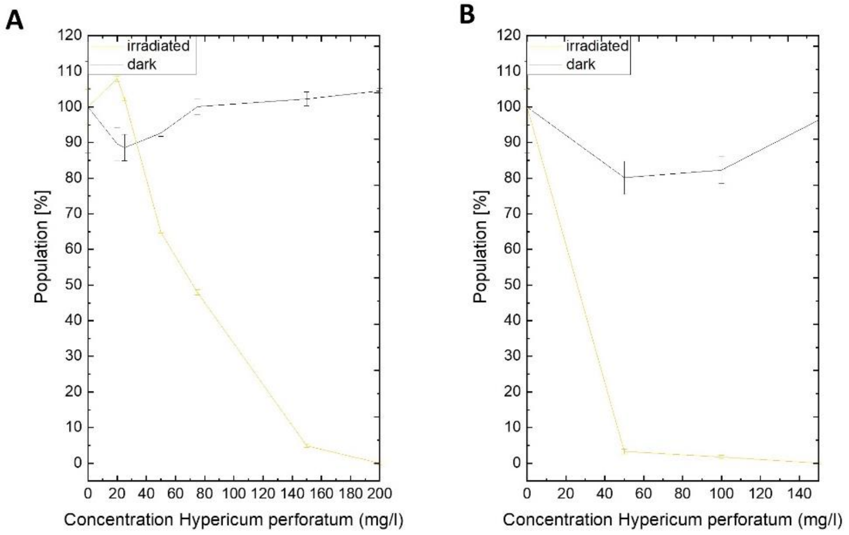

Figure S5. Dose-response curve of HP against A) *C. albicans* and B) *S. aureus*. All MOs were preincubated for PI = 10 min and irradiated with orange light (λ = 598 nm, H = 9.3 J/cm^2).

2.4.1 Dose-response curves of irradiation with red-light

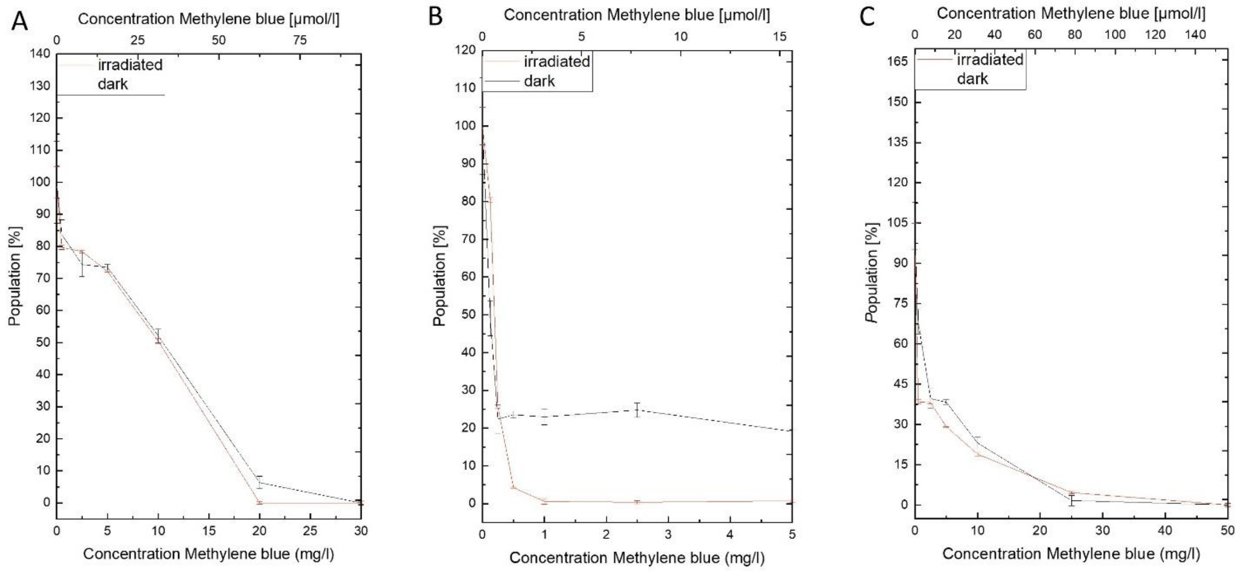

Figure S6. Dose-response curve of MB against A) *E. coli*, B) *C. albicans* and C) *S. aureus*. All MOs were preincubated for PI = 10 min and irradiated with red light (λ = 630 nm, H = 30 J/cm^2).

3 Mycochemical Part

3.1 Mycochemical Fingerprint of Extracts

3.1.1 UV-Vis Spectroscopic Investigations

Each fungal extract (m = 0.5 mg) was solved in methanol (V = 1 mL). After vortexing, the UV-Vis absorption spectra of the solutions were measured (λ = 200 – 800 nm) in a glass cuvette (Hellma type 100 – Macro cells, quartz glass, light pass l = 10 mm).

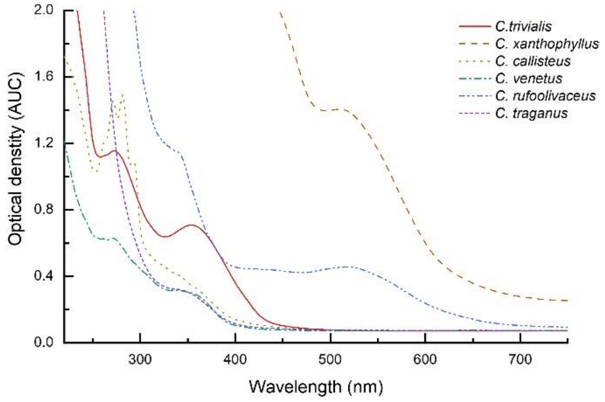

Figure S 7. UV-VIS absorption spectra of the investigated extracts (0.5 mg/ml, Methanol).

3.1.2 HPLC-DAD-ELSD/FLD/MS Analysis

All prepared extracts were solved in DMSO (c = 1 mg/ml) and filtered through cotton wool prior to analysis. The setting of the HPLC-DAD was kept constant (Table S1), while the parameters for the other hyphenated detectors (i.e., ELSD, FLD, MS) were changed individually. The mobile phases equaled water (A) and acidified acetonitrile (ACN + 0.1% formic acid (FA), B). As stationary phase, a Synergi MAX-RP (80Å, 4 µm, 150 x 4.60 mm) column was used. The injection volume was V = 5 µl. The flow rate was set to Q = 0.5 mL/min. Column temperature was set to T = 35°C.

Table S2. Solvent gradient used for the HPLC-DAD-X analysis

Time [min]	Solvent A [%] H_2O	Solvent B [%] ACN + 0.1% FA
0.00	90	10.0
40.0	10	90.0
50.0	2.0	98.0
51.0	90.0	10.0
55.0	90.0	10.0

As second detector, a fluorescence detector (FLD, Figure S8A, λ_{exc} = 455 nm, λ_{em} = 530 nm), an evaporative light scattering detector (ELSD, Figure S8B), or a mass spectrometer (MS, data not shown) were employed. In Figure S9 the results of the DAD measurement at λ = 254 nm are depicted. Figure S10 displays the results of the DAD measurement at λ = 428, 478, 528, and 598 nm.

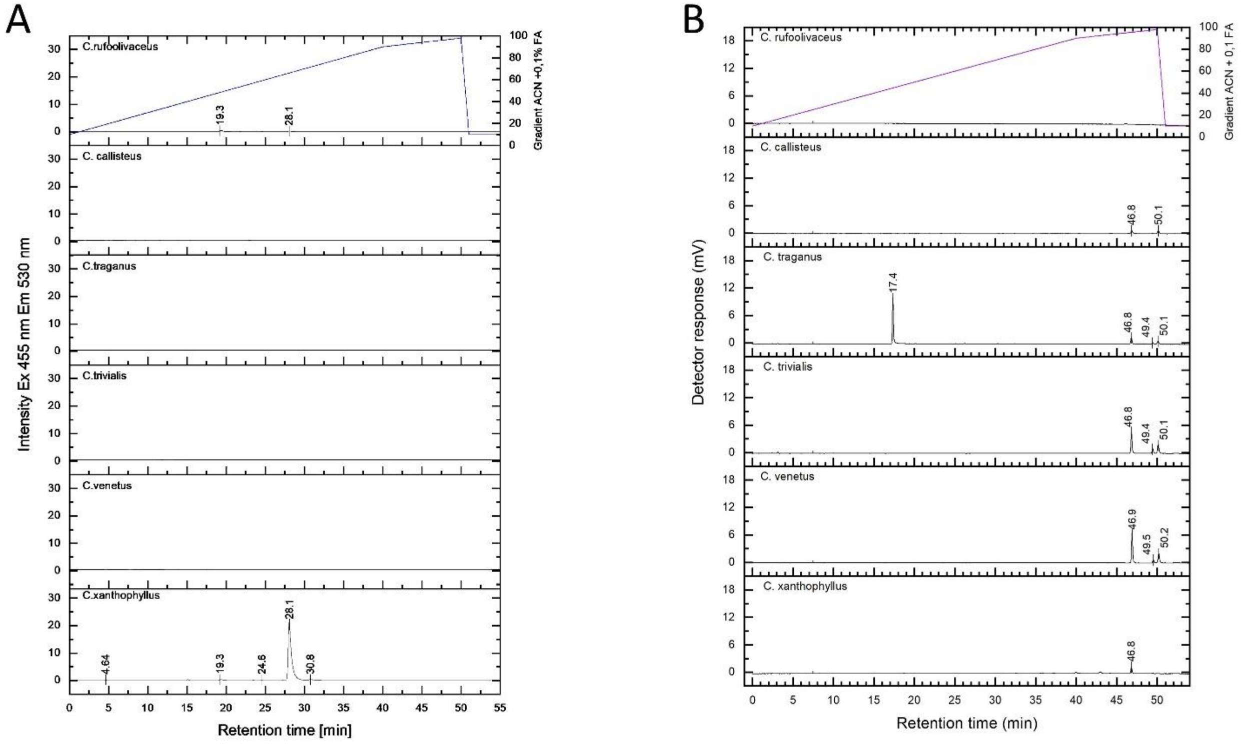

Figure S8 HPLC-FLD (A) and HPLC-ELSD (B) analysis of the six different extracts. The blue graph represent percent of ACN (+0.1% FA) in the mobile phase (H2O/(ACN+0.1%FA)).

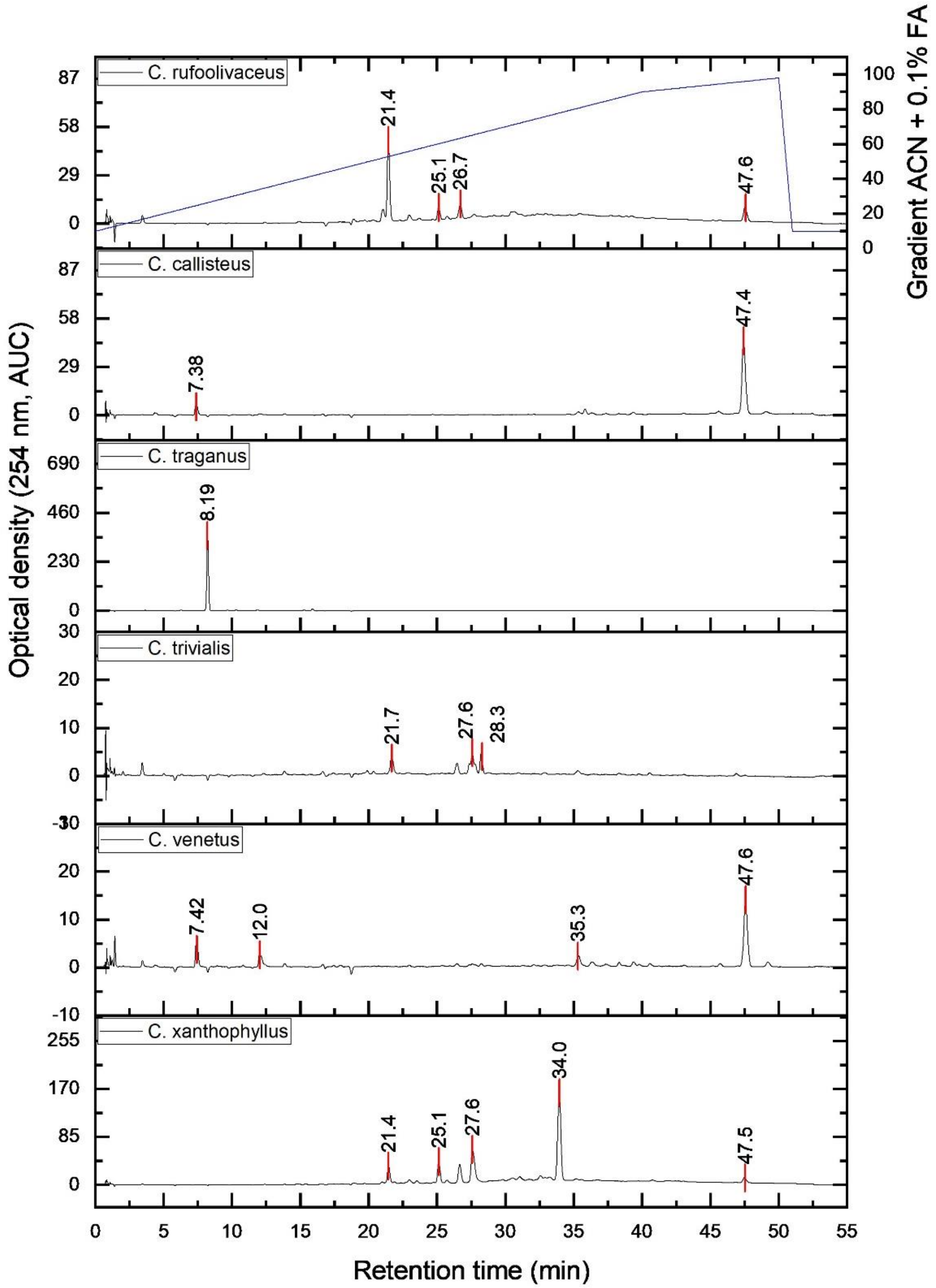

Figure S9. HPLC-DAD analysis of the six different extracts (c = 10 mg/ml) detected at λ = 254 nm. The blue graph represent percent of ACN (+0.1% FA) in the mobile phase (H_2O/(ACN+0.1%FA)).

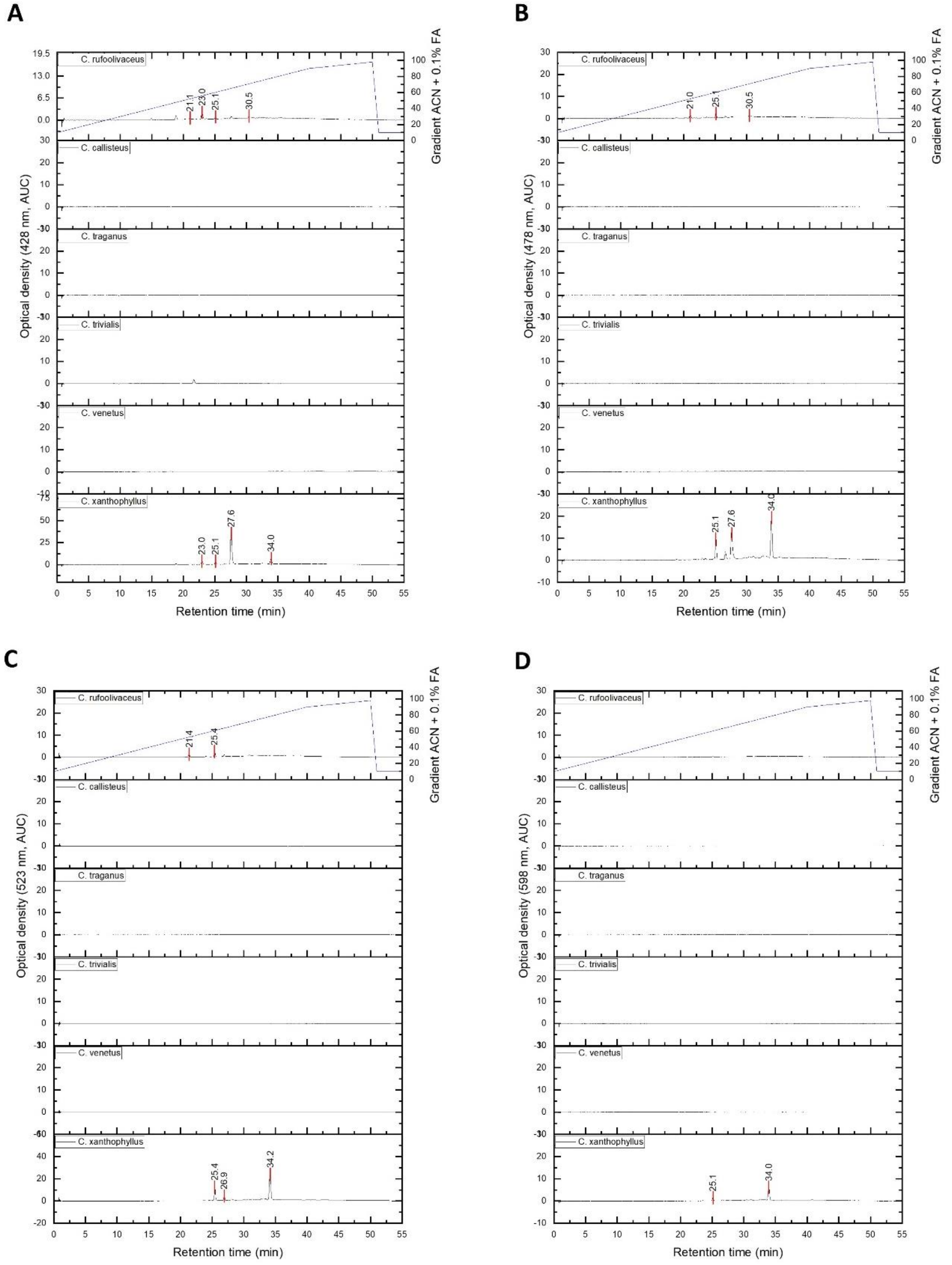

Figure S10. HPLC-DAD analysis of the six different extracts (c = 10 mg/ml). Detection wavelength A) 428 nm, B) λ = 478 nm, C) λ = 523 nm, D) λ = 598 nm. The blue graph represent percent of ACN (+0.1% FA) in the mobile phase (H_2O/(ACN+0.1%FA)).

3.1.3 General mycochemical analysis

The recorded FLD chromatograms (excitation wavelength of λ = 455 nm and emission wavelength of λ = 530 nm) of most species showed no significant peak. For *C. xanthophyllus*, however, one major peak (t_r = 28.1 min) was detected and correlated to Peak 4 (Figure S10A, t_{ret} = 27.6 min, Table S3). In traces this peak was also detected in *C. rufoolivaceus*. Furthermore, minor peaks were detected at t_r = 19.3 min for both extracts. In addition, for *C. xanthophyllus* two additional peaks at t_r = 24.6 and 30.8 min were observed.

The peak in the ELSD chromatogram (Fig S2B) at t_r = 46.8 min could be detected in all six extracts. It corresponded to a m/z of 279 $[M-H]^-$ in the HPLC-MS experiment and was putatively assigned as linoleic acid. The latter was found as major fatty acid in extracts of the related *Cortinarius magellanicus* (Toledo et al., 2016). Next to this peak, some minor peaks were detected with a similar retention time (t_r = 49.2 and 50.1 min) in several extracts. Furthermore, only in *C. traganus* a peak was detected by the ELSD detector with a retention time of t_r = 17.3 min, indicating a more polar compound.

3.1.4 Mycochemical analysis of *C. xanthophyllus*

The extract of *C. xanthophyllus* was characterized in the HPLC-DAD chromatogram by one major and four minor peaks which absorb at 254 nm. In detail, the major peak is peak **5** with a retention time of t_r = 34.2 min. The minors are peak **1-4** with a retention times of t_r = 21.7, 25.4, 26.9, and 28.0 minutes, respectively. The UV-Vis spectrum of each peak is depicted in Figure S12 and revealed that two anthraquinones with a λ_{max} < 500 nm and three anthraquinones with a λ_{max} > 500 nm contribute to the overall pigmentation of *C. xanthophyllus*. By the means of a HPLC-DAD-MS experiment the molecular weight of each peak was determined (Table S3).

Table S3. Tentative annotation of the pigments from *C. xanthophyllus*

	t_r [min]	Intensity (λ=254nm)	$[M-H]^-$	$[M+H]^+$	λ_{max} [nm]	Proposed Chemical Formula	Suggested Compound
1	21.7	Minor	645.6 (100%)	243.1 (26%), 261.1 (100%), 543.2 (15%)	210	-	n.d.
2	25.4	Minor	555.3 (100%)	557.2 (100%)	216, 300, 514	$C_{32}H_{28}O_9$	Rufoolivacin A
3	26.9	Minor	-	498.4 (23%) 519.2 (16%) 557.2 (100%)	298, 335, 493	$C_{32}H_{28}O_9$	Rufoolivacin C
4	28.0	Minor	608.8 (25%) 624.9 (100%)	285.1 (7%) 377.4 (6%) 474.5 (10%) 543.2 (100%) 585.2 (23%)	222, 270, 288, 436	$C_{16}H_{12}O_5$	Physcion/Parietin
5	34.2	Major	-	256.3 (9%) 409.4 (10%) 459.4 (10%) 501.4 (10%) 556.2 (100%)	228, 258, 335, 525	-	n.d.

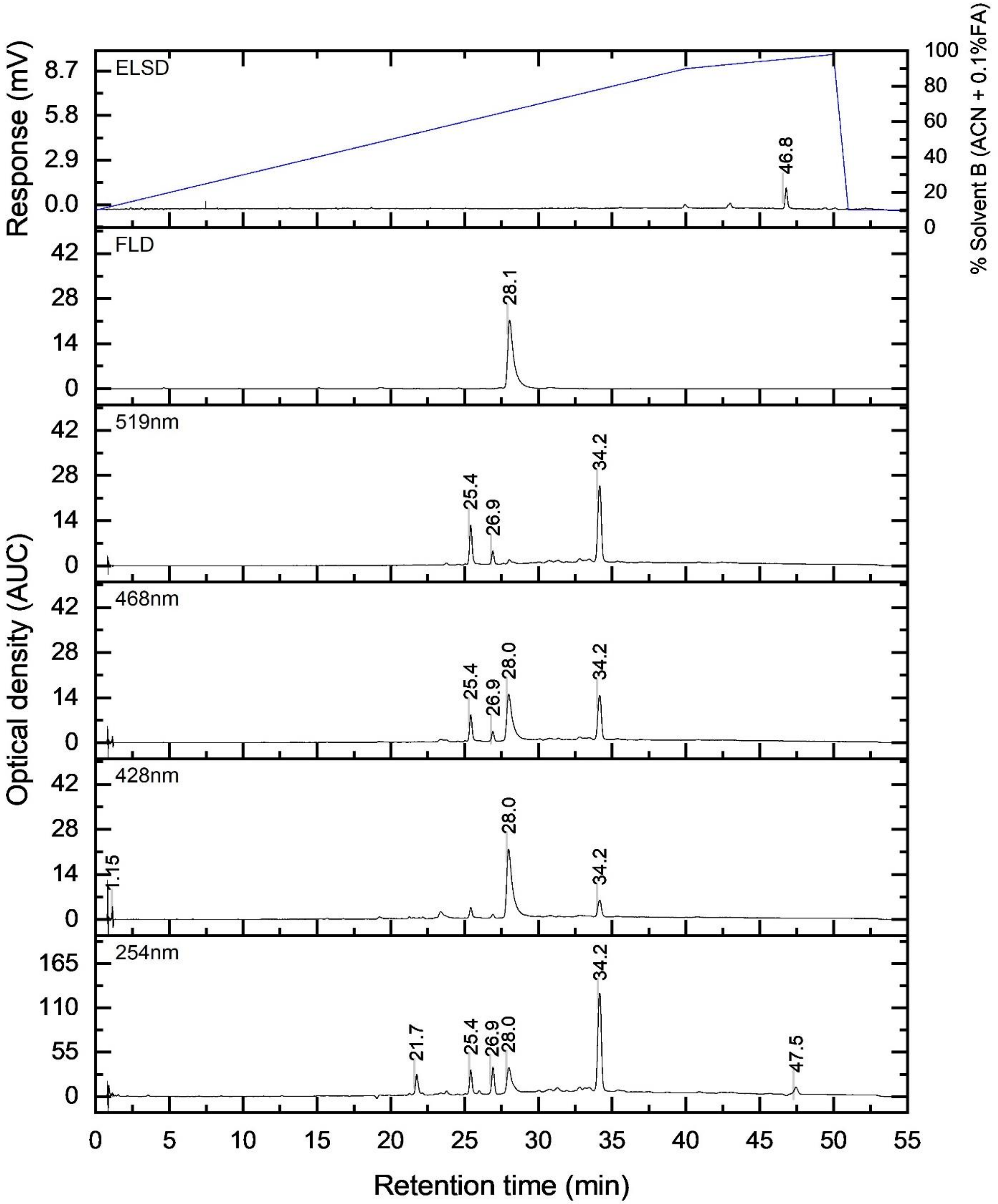

Figure S11. HPLC-DAD-X Chromatograms of the *C. xanthophyllus* extract (1 mg/ml, DMSO): From the bottom to the top the chromatograms detected at λ = 254 nm, 428 nm, 468 nm, 519 nm are depicted as well as the FLD (λ_{exc} = 455 nm, λ_{det} = 530 nm) and ELSD chromatogram.

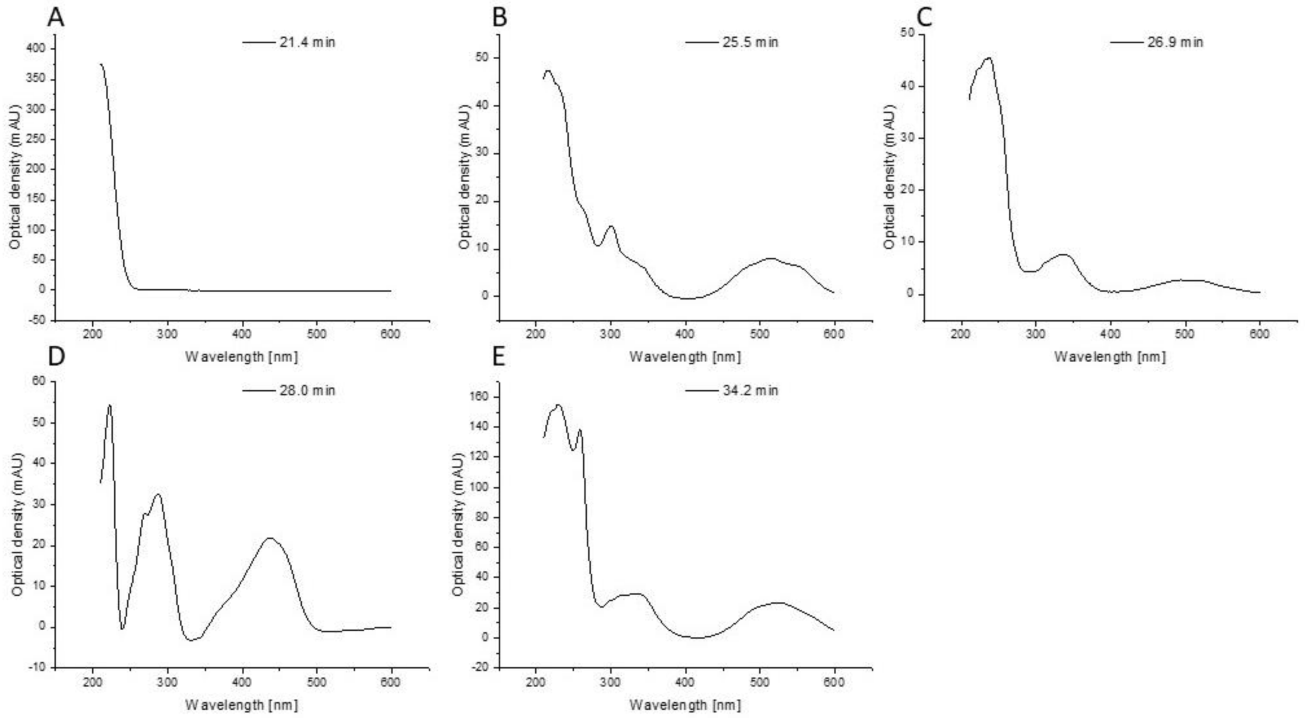

Figure S12: UV-Vis spectra of the major and minor peaks of *C. xanthophyllus* extracted from the HPLC-DAD experiment. Peaks are sorted according to their retention time, A) t_r = 21.4 min, B) t_r = 25.5 min, C) t_r = 29.9 min, D) t_r = 28.0 min, E) t_r = 34.2 min.

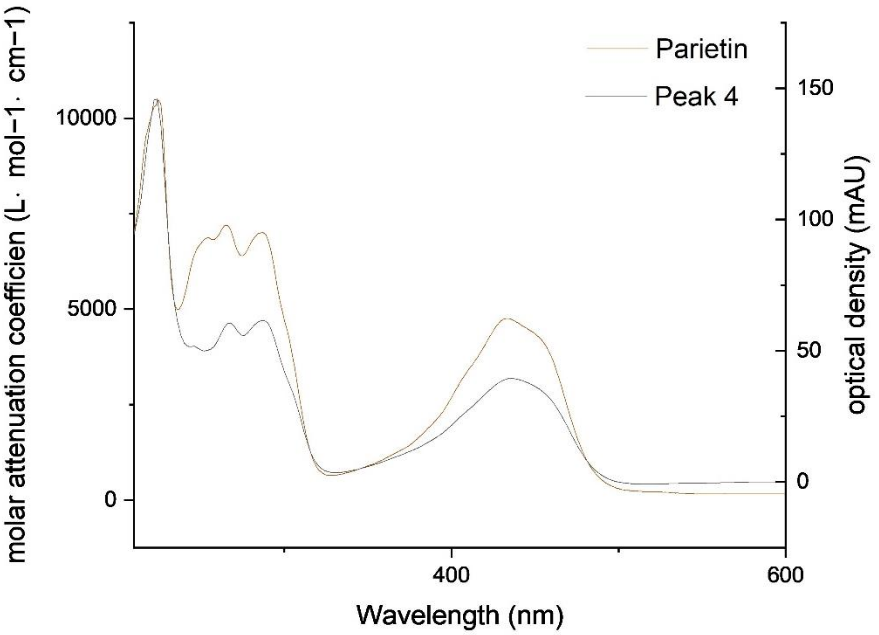

Figure S13: UV-VIS overlay of parietin (reference compound) and peak **4** of the *C. xanthophyllus* extract. The minor differences can be attributed to the different solvents used (i.e., MeOH and ACN+0.1%FA, respectively).

4 (Photo)antimicrobial evaluation of the *C. xanthophyllus* extract

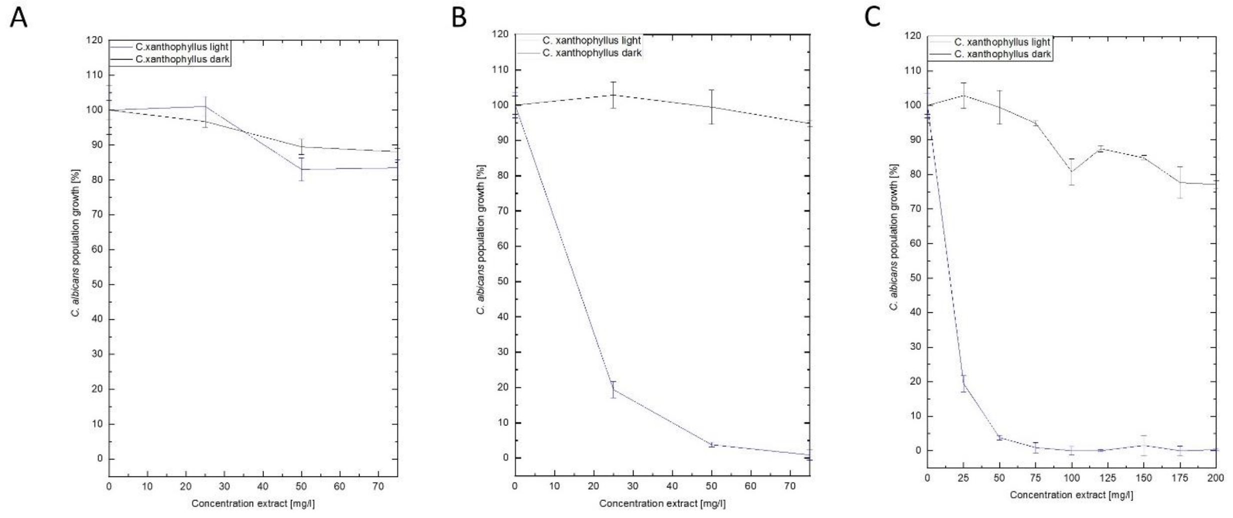

Figure S14. (Photo)antimicrobial action of *C. xanthophyllus* extract against *C. albicans* under blue light irradiation (λ = 468 nm, H = 30 J/cm^2) in dependency of the pre-incubation time. A) PI = 10 min, B) PI = 60 min, C) PI = 60 min and an extended concentration range showing a large therapeutic window.

5 References for Supplementary Material

Alam, S.T., Le, T.a.N., Park, J.-S., Kwon, H.C., and Kang, K. (2019). Antimicrobial Biophotonic Treatment of Ampicillin-Resistant Pseudomonas aeruginosa with Hypericin and Ampicillin Cotreatment Followed by Orange Light. *Pharmaceutics* 11**,** 641.

Bauer, P. (2016). Photodynamische Inaktivierung von Mikroorganismen mittels Perinaphthenonderivaten – Einfluss einer LED405nm-Lampe. *Inaugural - Dissertation, Fakultät für Medizin der Universität Regensburg* 1.

Bhavya, M.L., and Umesh Hebbar, H. (2019). Efficacy of blue LED in microbial inactivation: Effect of photosensitization and process parameters. *Int J Food Microbiol* 290**,** 296-304.

Bresolí-Obach, R., Gispert, I., Peña, D.G., Boga, S., Gulias, Ó., Agut, M., Vázquez, M.E., and Nonell, S. (2018). Triphenylphosphonium cation: A valuable functional group for antimicrobial photodynamic therapy. *Journal of Biophotonics* 11**,** e201800054.

Carmello, J.C., Dovigo, L.N., Mima, E.G., Jorge, J.H., De Souza Costa, C.A., Bagnato, V.S., and Pavarina, A.C. (2015). In vivo evaluation of photodynamic inactivation using Photodithazine(R) against Candida albicans. *Photochem Photobiol Sci* 14**,** 1319-1328.

Carmello, J.C., Dovigo, L.N., Mima, E.G., Jorge, J.H., De Souza Costa, C.A., Bagnato, V.S., and Pavarina, A.C. (2017). Correction: In vivo evaluation of photodynamic inactivation using Photodithazine(R) against Candida albicans. *Photochem Photobiol Sci* 16**,** 1336-1337.

Carvalho, G.G., Felipe, M.P., and Costa, M.S. (2009). The photodynamic effect of methylene blue and toluidine blue on Candida albicans is dependent on medium conditions. *J Microbiol* 47**,** 619-623.

Da Collina, G.A., Freire, F., Santos, T., Sobrinho, N.G., Aquino, S., Prates, R.A., Da Silva, D.F.T., Tempestini Horliana, A.C.R., and Pavani, C. (2018). Controlling methylene blue aggregation: a more efficient alternative to treat Candida albicans infections using photodynamic therapy. *Photochem Photobiol Sci* 17**,** 1355-1364.

De Oliveira, E.F., Tosati, J.V., Tikekar, R.V., Monteiro, A.R., and Nitin, N. (2018). Antimicrobial activity of curcumin in combination with light against Escherichia coli O157:H7 and Listeria innocua : Applications for fresh produce sanitation. *Postharvest Biology and Technology* 137**,** 86-94.

Delcanale, P., Hally, C., Nonell, S., Bonardi, S., Viappiani, C., and Abbruzzetti, S. (2020). Photodynamic action of Hypericum perforatum hydrophilic extract against Staphylococcus aureus. *Photochem Photobiol Sci* 19**,** 324-331.

Dovigo, L.N., Pavarina, A.C., Carmello, J.C., Machado, A.L., Brunetti, I.L., and Bagnato, V.S. (2011). Susceptibility of clinical isolates of Candida to photodynamic effects of curcumin. *Lasers Surg Med* 43**,** 927-934.

Flors, C., and Nonell, S. (2006). Light and Singlet Oxygen in Plant Defense Against Pathogens: Phototoxic Phenalenone Phytoalexins. *Accounts of Chemical Research* 39**,** 293-300.

Gunics, G., Motohashi, N., Amaral, L., Farkas, S., and Molnár, J. (2000). Interaction between antibiotics and non-conventional antibiotics on bacteria. *International Journal of Antimicrobial Agents* 14**,** 239-242.

Hosseini, N., Yazdanpanah, S., Saki, M., Rezazadeh, F., Ghapanchi, J., and Zomorodian, K. (2016). Susceptibility of Candida albicans and Candida dubliniensis to Photodynamic Therapy Using Four Dyes as the Photosensitizer. *J Dent (Shiraz)* 17**,** 354-360.

Ilizirov, Y., Formanovsky, A., Mikhura, I., Paitan, Y., Nakonechny, F., and Nisnevitch, M. (2018). Effect of Photodynamic Antibacterial Chemotherapy Combined with Antibiotics on Gram-Positive and Gram-Negative Bacteria. *Molecules* 23.

Jiang, Y., Leung, A.W., Hua, H., Rao, X., and Xu, C. (2014). Photodynamic Action of LED-Activated Curcumin againstStaphylococcus aureusInvolving Intracellular ROS Increase and Membrane Damage. *International Journal of Photoenergy* 2014**,** 1-7.

Muehler, D., Sommer, K., Wennige, S., Hiller, K.A., Cieplik, F., Maisch, T., and Späth, A. (2017). Light-activated phenalen-1-one bactericides: efficacy, toxicity and mechanism compared with benzalkonium chloride. *Future Microbiol* 12**,** 1297-1310.

Nisnevitch, M., Nakonechny, F., and Nitzan, Y. (2010). Photodynamic antimicrobial chemotherapy by liposome-encapsulated water-soluble photosensitizers. *Bioorg Khim* 36**,** 396-402.

Pasyechnikova, N., Zborovskaya, O., and Kustrin, T. (2009). In vitro photodynamic properties of methylene blue in a combination with laser illumination at 630 nm concerning Candida albicans. *Klin Oczna* 111**,** 15-17.

Peloi, L.S., Soares, R.R., Biondo, C.E., Souza, V.R., Hioka, N., and Kimura, E. (2008). Photodynamic effect of light-emitting diode light on cell growth inhibition induced by methylene blue. *J Biosci* 33**,** 231-237.

Penha, C.B., Bonin, E., Da Silva, A.F., Hioka, N., Zanqueta, É.B., Nakamura, T.U., De Abreu Filho, B.A., Campanerut-Sá, P.a.Z., and Mikcha, J.M.G. (2017). Photodynamic inactivation of foodborne and food spoilage bacteria by curcumin. *LWT - Food Science and Technology* 76**,** 198-202.

Rossoni, R.D., Junqueira, J.C., Santos, E.L., Costa, A.C., and Jorge, A.O. (2010). Comparison of the efficacy of Rose Bengal and erythrosin in photodynamic therapy against Enterobacteriaceae. *Lasers Med Sci* 25**,** 581-586.

Tabenski, I., Cieplik, F., Tabenski, L., Regensburger, J., Hiller, K.A., Buchalla, W., Maisch, T., and Späth, A. (2016). The impact of cationic substituents in phenalen-1-one photosensitizers on antimicrobial photodynamic efficacy. *Photochem Photobiol Sci* 15**,** 57-68.

Thesnaar, L., Bezuidenhout, J.J., Petzer, A., Petzer, J.P., and Cloete, T.T. (2021). Methylene blue analogues: In vitro antimicrobial minimum inhibitory concentrations and in silico pharmacophore modelling. *Eur J Pharm Sci* 157**,** 105603.

Toledo, C., Barroetaveña, C., Fernandes, A., Barros, L., and Ferreira, I. (2016). Chemical and Antioxidant Properties of Wild Edible Mushrooms from Native Nothofagus spp. Forest, Argentina. *Molecules* 21**,** 1201.

Torres-Hurtado, S.A., Ramirez-Ramirez, J., Larios-Morales, A.C., Ramirez-San-Juan, J.C., Ramos-Garcia, R., Espinosa-Texis, A.P., and Spezzia-Mazzocco, T. (2019). Efficient in vitro photodynamic inactivation using repetitive light energy density on Candida albicans and Trichophyton mentagrophytes. *Photodiagnosis Photodyn Ther* 26**,** 203-209.

Wainwright, M., and Crossley, K.B. (2002). Methylene Blue--a therapeutic dye for all seasons? *J Chemother* 14**,** 431-443.

Wainwright, M., Phoenix, D.A., Gaskell, M., and Marshall, B. (1999). Photobactericidal activity of methylene blue derivatives against vancomycin-resistant Enterococcus spp. *J Antimicrob Chemother* 44**,** 823-825.

Wainwright, M., Phoenix, D.A., Laycock, S.L., Wareing, D.R., and Wright, P.A. (1998). Photobactericidal activity of phenothiazinium dyes against methicillin-resistant strains of Staphylococcus aureus. *FEMS Microbiol Lett* 160**,** 177-181.

Wainwright, M., Phoenix, D.A., Marland, J., Wareing, D.R., and Bolton, F.J. (1997). A study of photobactericidal activity in the phenothiazinium series. *FEMS Immunol Med Microbiol* 19**,** 75-80.
